# Artificial intelligence in commercial fracture detection products: a systematic review and meta-analysis of diagnostic test accuracy

**DOI:** 10.1038/s41598-024-73058-8

**Published:** 2024-10-04

**Authors:** Julius Husarek, Silvan Hess, Sam Razaeian, Thomas D. Ruder, Stephan Sehmisch, Martin Müller, Emmanouil Liodakis

**Affiliations:** 1 Department of Orthopaedic Surgery and Traumatology, Bern University Hospital, Inselspital, University of Bern, Bern, Switzerland; 2 University of Bern, Bern, Switzerland; 3 Faculty of Medicine, Medical University of Sofia, Sofia, Bulgaria; 4 Department for Trauma, Hand and Reconstructive Surgery, Saarland University, Kirrberger Str. 100, 66421 Homburg, Germany; 5 Interventional and Pediatric Radiology, Inselspital, Bern University Hospital, University Institute of Diagnostic, University of Bern, Bern, Switzerland; 6 Department of Trauma Surgery, Hannover Medical School, Carl-Neuberg-Straße 1, 30625 Hannover, Germany; 7 Department of Emergency Medicine, Bern University Hospital, Inselspital, University of Bern, Bern, Switzerland

**Keywords:** Bone imaging, Fracture repair

## Abstract

Conventional radiography (CR) is primarily utilized for fracture diagnosis. Artificial intelligence (AI) for CR is a rapidly growing field aimed at enhancing efficiency and increasing diagnostic accuracy. However, the diagnostic performance of commercially available AI fracture detection solutions (CAAI-FDS) for CR in various anatomical regions, their synergy with human assessment, as well as the influence of industry funding on reported accuracy are unknown. Peer-reviewed diagnostic test accuracy (DTA) studies were identified through a systematic review on Pubmed and Embase. Diagnostic performance measures were extracted especially for different subgroups such as product, type of rater (stand-alone AI, human unaided, human aided), funding, and anatomical region. Pooled measures were obtained with a bivariate random effects model. The impact of rater was evaluated with comparative meta-analysis. Seventeen DTA studies of seven CAAI-FDS analyzing 38,978 x-rays with 8,150 fractures were included. Stand-alone AI studies (*n* = 15) evaluated five CAAI-FDS; four with good sensitivities (> 90%) and moderate specificities (80–90%) and one with very poor sensitivity (< 60%) and excellent specificity (> 95%). Pooled sensitivities were good to excellent, and specificities were moderate to good in all anatomical regions (*n* = 7) apart from ribs (*n* = 4; poor sensitivity / moderate specificity) and spine (*n* = 4; excellent sensitivity / poor specificity). Funded studies (*n* = 4) had higher sensitivity (+ 5%) and lower specificity (-4%) than non-funded studies (*n* = 11). Sensitivity did not differ significantly between stand-alone AI and human AI aided ratings (*p* = 0.316) but specificity was significantly higher the latter group (*p* < 0.001). Sensitivity was significant lower in human unaided compared to human AI aided respectively stand-alone AI ratings (both *p* ≤ 0.001); specificity was higher in human unaided ratings compared to stand-alone AI (*p* < 0.001) and showed no significant differences AI aided ratings (*p* = 0.316). The study demonstrates good diagnostic accuracy across most CAAI-FDS and anatomical regions, with the highest performance achieved when used in conjunction with human assessment. Diagnostic accuracy appears lower for spine and rib fractures. The impact of industry funding on reported performance is small.

**Supplementary Information:**

The online version contains supplementary material available at 10.1038/s41598-024-73058-8.

## Introduction

Bone fractures are a significant global public health burden and one of the main reasons for emergency department (ED) visits^1^. In a comprehensive prospective fracture database, an estimated overall incidence of 1fractures per 100,000 people per year was documented, leading to an annual fracture incidence rate of 1.2%, with an increase from the age of 50 up to 8% ^2^. An estimate from 2005 predicted a drastic increase of up to 50% in the annual incidence of fractures by 2025 due to an aging population^3^.

Radiological imaging is an essential component of the diagnostic process in ED, accounting for over 20% of all emergency resources utilized^4^. The number of radiologic examinations performed per patient in the ED has continued to disproportionately increase when compared to the consistently rising number of patient consultations: since 2006, there has been a doubling in the number of conventional radiography (CR) images and computed tomography (CT) examinations have witnessed a staggering 700% increase during this timeframe^5^.

The rising incidence of fractures in an aging population and the disproportionate increase in radiological diagnostics have led to various problems in everyday clinical practice: Firstly, the high workload during shift work can lead to stress and medical errors^6–8^. Secondly, prioritized CT scan examination may lead to a delayed release of final CR reports, resulting in discrepancies with the emergency physician’s preliminary discharge letter and can cause clinically relevant diagnostic errors in up to 7.5% of cases^9^. Thirdly, the desired communication between various medical disciplines is frequently insufficient, primarily relying on telephone interactions. This limited interaction can impede the joint review of imaging examinations and potentially lead to false-negative findings in cases where inexperienced emergency physicians lack adequate support from experienced radiologists^10^.

Artificial intelligence (AI) seems to be a promising approach to address these issues and to assist clinicians, especially during radiographic fracture detection. In this context, certified and commercially available AI fracture detection solutions (CAAI-FDS) are of special interest, as they are expected to meet regulatory requirements (e.g., data privacy) and quality standards, as well as safety criteria, along with higher accuracy and reliability.

Initial studies reported a time saving of between 6.3 and 11.6 s per interpreted CR with the support of AI, which may help to reduce workload and stress levels throughout the day^11–13^. Furthermore, studies have shown that AI can provide several solutions with promising results in the detection of fractures, and may lead to a reduction in diagnostic errors^14–17^.

These reviews and diagnostic test accuracy (DTA) meta-analyses have several short-comings: Up to now, most analyses have focused on proof-of-concept studies. However, a dynamic advancement has led to a rapid increase in peer-reviewed studies evaluating the diagnostic accuracy of CAAI-FDS. Additionally, previous overviews dealing with the use of commercially AI solutions in radiodiagnostics have neglected one crucial aspect – a thorough evaluation of the diagnostic accuracy of the products presented to address and clarify relevant medical and legal aspects. Currently, there is a gap in the existing literature as no comparative meta-analysis has been conducted to evaluate the diagnostic performance of different types of raters (stand-alone AI vs. human without AI assistance vs. human with AI assistance). Furthermore, there are no stratified analyses examining different anatomical regions, which makes it difficult to identify local performance limitations of CAAI-FDS. The impact of industry funding of trials on reported diagnostic accuracy is also unexplored, opening the possibility of reporting bias as only 6% of scientific analyses of AI products are conducted independently, resulting in potential bias regarding the evidence and a lack of transparency^16,18^. Lastly, there is a lack of investigation of the reference standards used to validate the diagnostic accuracy of both AI and human raters, making a comprehensive investigation essential to ensure reliability and comparability.

Moreover, despite the growing availability, identifying the most suitable product in this rapidly expanding market remains a challenging endeavor, given that studies have unveiled significant variations in diagnostic accuracy^16,18,19^.

Thus, the objectives of this DTA systematic review and meta-analysis were:

i. Provide a current overview of FDA-cleared and CE-marked CAAI-FDS based on CR whose diagnostic accuracy had been validated by peer-reviewed publications.
ii. Systematically evaluate and compare the diagnostic accuracy of identified CAAI-FDS, employing standardized performance metrics. Quantify the diagnostic performance according to different anatomical regions, industry funding and the chosen reference standard.
iii. Evaluation of the added value of CAAI-FDS by comparing the diagnostic accuracy of human ratings with and without assistance, as well as stand-alone AI ratings.

## Methods

This study was conducted in accordance with the Cochrane Handbook for Systematic Reviews of DTA^20^ and reported by the Preferred Reporting Items for a Systematic Review and Meta-analysis of Diagnostic Test Accuracy Studies (PRISMA-DTA)^21^. The comprehensive study consists of two different parts. First, identification of FDA-cleared and CE-marked CAAI-FDS and their corresponding companies. Second, a systematic review and meta-analysis to identify peer-reviewed publications and evaluating DTA for the identified CAAI-FDS.

### Part one – product identification

The independent web-based overview from the Diagnostic Image Analysis Group (DIAG) at Radboud University Medical Center in the Netherlands^22^ and the American College of Radiology Data Science Institute (ACR DSI) AI Central database^23^ were used for company and product identification. The DIAG maintains a website that offers information on AI-based products for clinical radiology practice, with a focus on CE-marked products for the European market, whereas the ACR DSI AI Central database provides information for products that have received clearance from the FDA, with a specific focus on the US market.

The DIAG and ACR DSI were searched (August 29th, 2023, updated November 11th, 2023) using search filters within the databases for CAAI-FDS. All selected companies and the products offered were checked for their current approval status and exact function for clinical use.

### Criteria for product eligibility

1. Operation based on CR images.
2. CE-marked or FDA-cleared approval status.
3. Availability as a commercially marketed product for clinical use focusing on fracture detection.

### Part two – systematic review

First, all identified companies and product names identified in part one were connected with the Boolean operator ‘OR’ for a PubMed and Embase database search (November 11th, 2023) via their corresponding website engine to identify DTA studies for meta-analysis. To increase the sensitivity of the search, CAAI-FDS that work with CT images were also included in the search string. The search syntax is shown in Supplement III.

Second, the Citationchaser tool^24^ was utilized for the found publications on the company’s websites.

### Criteria for final study eligibility

The final study eligibility criteria were based on the PICO scheme:

Population: Patients with CR that were investigated for the presence of any kind of fracture with a defined reference standard as ground truth.

Intervention: Detection by any type of CAAR-FDS or with its assistance.

Comparison: All comparisons.

Outcomes: Values for sensitivity and specificity with 95% confidence interval (CI) and/or true positives (TP), false positives (FP), false negatives (FN), and true negatives (TN).

Furthermore, the publications should be in English and dated 2015 or later with a peer reviewed status. Mention of the product name in the publication or mention of the publication on the company’s website that allows a direct link to the CAAI-FDS. Studies that did not meet the above criteria were excluded.

### Screening process

Results of the different databases and from the citation chasing were collected and checked for duplications based on the method described by Bramer et al.^25^ using EndNote 20.5 (Clarivate Analytics, PA, USA). The remaining articles were entered into the web-based platform Rayyan^26^ for an accurate screening process. Subsequently, two authors (J.H. and S.H.) conducted independent reviews of both the title and abstract, followed by a comprehensive evaluation of the full text of the remaining studies if deemed appropriate. Discrepancies were discussed between the authors and resolved by consensus.

### Data collection process

One author (J.H.) extracted the values for TP, FP, FN, and TN for all subgroups directly out of the included studies, or calculated based on the given information. If only the sensitivity with 95% confidence interval (CI) and specificity with 95% CI was given, the Wald interval error formula^27^ was used to estimate TP, FP, FN, and TN (see Supplement I). Further missing information or data was requested from the corresponding authors. These data were entered into a predefined collection form using Microsoft Excel 16.6 (Microsoft Corporation, WA, USA). A second author (S.H.) then reviewed the collection by comparing the data sheet with the information from the included studies. Any ambiguities and differences of interpretation were discussed and resolved by mutual agreement or with the help of a statistical expert (M.M.).

In addition to these values, detailed information was collected for each article identified and included in the study. This included the author’s name, year of publication, characteristics of the population (e.g. sample size, age structure, gender distribution), assessment characteristics of diagnostic accuracy (e.g. stand-alone AI, unaided or aided human assessment), type of CAAI-FDS used, reference standards used, and financial sources.

### Risk of bias and applicability

The Quality Assessment of Diagnostic Accuracy Studies (QUADAS-2)^28^ recommended by the Cochrane Collaboration was used to evaluate the risk of bias (RoB). A modified version of the suggested list of signaling questions was created for each domain adapted to this systematic review. The complete list of signaling questions is available in the Supplement IV. Four domains included patient selection, index test, reference standard and funding were qualified with the support of the signaling questions as ‘low’, ‘high’ or ‘unclear’ for risk of bias and applicability. The assessments were performed independently by two authors (J.H. and S.H.). Disagreements were resolved by discussion. If the two researchers could not reach a consensus, a senior author was consulted to help to reach a final decision. Finaly, each included study was assessed for an overall rating. Publications that received one ‘high’ RoB rating were categorized as ‘moderate’ RoB studies, two or more ‘high’ RoB ratings were automatically categorized as ‘high’ RoB studies according to the QUADAS criteria.

### Data synthesis and statistical analysis

The statistical analysis followed the recommendations of the Cochrane Handbook for Systematic Reviews of DTA^20^ and was performed in STATA 18.1 (StataCorp, College Station, TX, USA) and the – *metadta* – command^29^ for meta-analysis and meta-regression of DTA data. The strength of this command is that it avoids lengthy and tedious calculations for fitting regression models and processing the estimates by bundling appropriate statistical procedures in a robust and user-friendly program and implementing state-of-the-art statistical methods for meta-analysis of DTA studies^29^. The command utilizes a generalized linear mixed model for the binomial category, employing a logit link as recommended by the Chap. 10 of the *Cochrane Handbook for Systematic Reviews of Diagnostic Test*^30^. It quantifies the between-study heterogeneity using the I^2^ statistics by Zhou & Dendukuri (2014)^31^. The generalized I^2^ is obtained by the random-effect bivariate model and is shown for every forest plot, it summarizes the overall heterogeneity, by considering the correlation between sensitivity and specificity. Furthermore, detailed statistics for the between-study heterogeneity for each forest plot are shown in Supplement V.

If the estimated numbers with the use of – *metadta* – command revealed the extracted values of sensitivity and specificity with 95% CI with a maximum absolute deviation of 1% the numbers were used, otherwise the estimates were not included in the meta-analysis.

If more than one diagnostic accuracy estimate was presented in a subgroup analysis for a study, all available estimates were pooled first by a random-effects meta-analysis and the pooled estimates were used in the further analysis.

For each analysis the associated forest plots are shown. Sensitivity and specificity are given as proportions with 95% CI. Sensitivity and specificity levels were grouped as very poor (< 0.60) poor (0.60 to < 0.70), moderate (0.70 to < 0.90), good (0.90 to < 0.95) and excellent (0.95 and above).

Different stratified analyses were performed (e.g. by CAAI-FDS, by body region, reference standard and industry funding status) with descriptive analysis of the pooled estimates, i.e. without statistical comparison of the pooled estimates. A comparative meta-analysis between the three rater groups (stand-alone AI vs. human unaided vs. human AI aided ratings) was performed for the studies that provided results for these three groups. Additionally, this analysis was restricted to unfunded studies. In comparative meta-analysis, the study-specific relative sensitivity and specificity with 95% CI was calculated additionally to the forest plot. A p-value of < 0.05 was defined as significant. No correction for multiple testing was performed.

### Code availability

The analytic code for STATA 18.1 (StataCorp, College Station, TX, USA) is uploaded as a Supplementary document.

## Results

### Product selection

In total, 214 general AI products with CE marking and 229 with FDA clearance were identified within the databases for commercial distribution. Of these, five CAAI-FDS were listed by DIAG with European market certification and four were listed in the ACR DSI database for the US market. Three CAAI-FDS are CE-marked and FDA-cleared. Finally, seven certified CAAI-FDS of six companies using CR were identified. Included CAAI-FDS are shown in Table 1.

**Table 1:** Descriptive characteristics of the included commercial fracture detection products for conventional radiography, sorted by company name.

Company	Founded	Main Office	Website	Product	Released	CE-certified	FDA-cleared
Annalise-AI	2019	Sydney, Australia	https://annalise.ai	Enterprise CXR Triage Trauma	2020	✓	✓
AZmed	2018	Paris, France	http://azmed.co	Rayvolve	2019	✓	✓
Imagen Technologies	2015	New York, USA	https://imagen.ai	OsteoDetect	2018	✗	✓
				FractureDetect	2020		
Milvue	2018	Paris, France	http://milvue.com	Suite - SmartUrgences	2020	✓	✗
Radiobotics	2017	Copenhagen, Denmark	http://radiobotics.com	RBfracture	2022	✓	✗
Gleamer	2017	Paris, France	http://gleamer.ai	BoneView Trauma	2020	✓	✓

*CE* Conformité Européenne, *FDA* Food and Drug Administration.

### Study selection

**Fig. 1:**
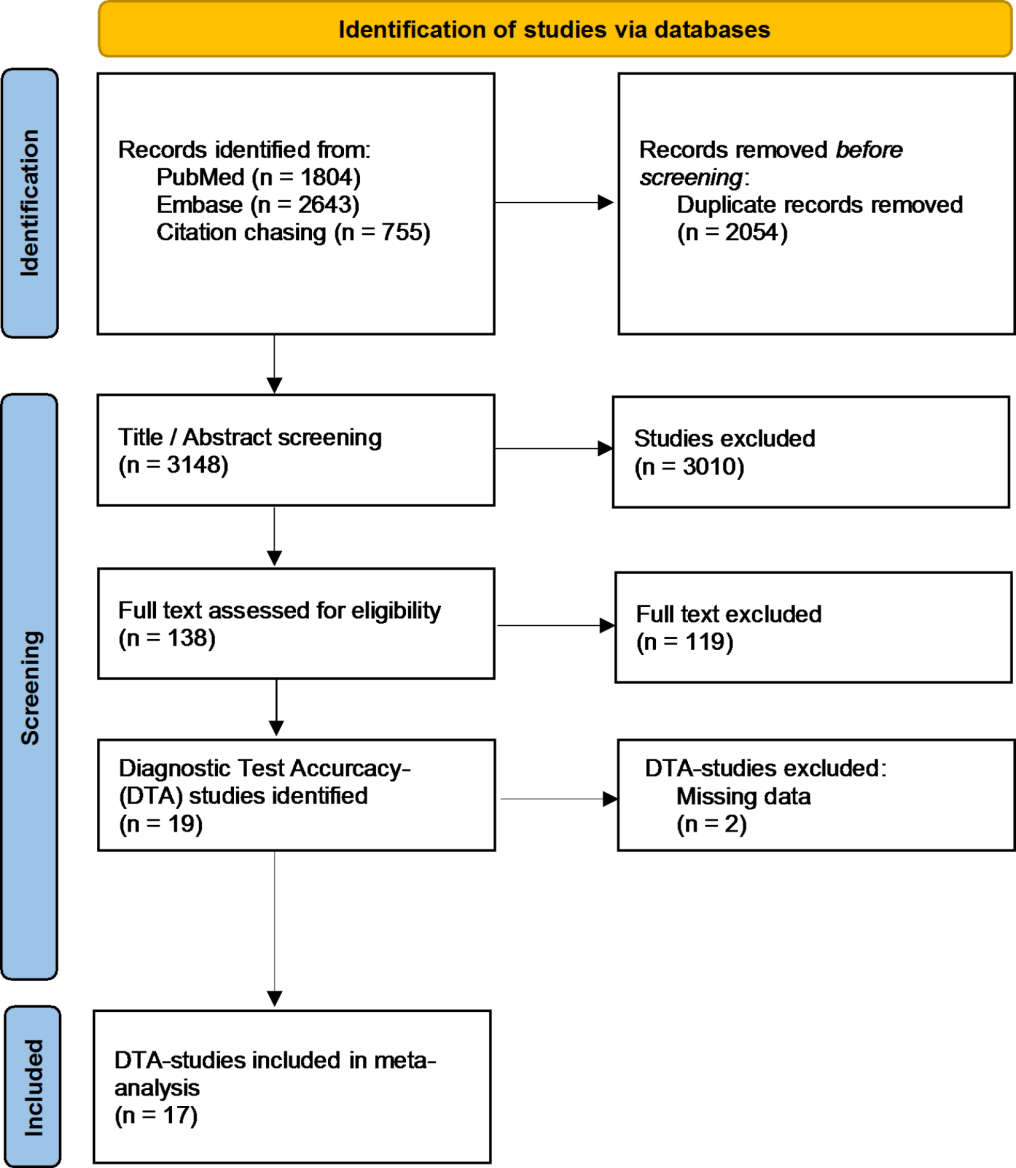
PRISMA flowchart.

The PubMed (*n* = 1804) and Embase (*n* = 2643) search resulted in a total of 4447 publications. Eighteen peer reviewed DTA studies were found on the respective company websites, which were subsequently used for citation chasing. In this way, a total of 755 forward and backward references were identified. After removal of all duplicates (*n* = 2054), 3148 publications were included for the title and abstract screening. After the first round of screening, 138 full texts were analyzed. Finally, 19 DTA publications were identified that showed a connection to CAAI-FDS based on CR. Two studies were excluded from the analysis due to missing data: The study by Shelmerdine et al.^32^ was excluded because it did not provide isolated sensitivity and specificity for fracture detection, as multiple pathologies were examined for CR. Lindsey et al.^33^ was excluded due to insufficient data on the total number of fractures, which prevented the calculation of a contingency table. Additionally, the determination by the Wald interval error formula^27^ resulted in insufficient deviations. Finaly, resulting in a total of 17 studies included in the DTA meta-analysis (see Fig. 1 flowchart). Descriptive characteristics of the selected studies are shown in Table 2.

**Table 2:** Descriptive characteristics of the included studies sorted by author.

Author	Product	Male	Female	Mean Age (Range)	Sample size	Fracture size	Risk of bias	Reference standard (based on)	Funding
Anderson et al.^36^	FractureDetect	73	102	N/A (22 to > 75)	4200	1008	Moderate	Expert consensus (CR)	✓
Bousson et al.^50^	SmartUrgence	742	468	41.3 (15 to 104)	1500	356	Low	Expert consensus (CR	✗
	BoneView								
	Rayvolve								
Canoni-Meynet et al.^13^	BoneView	268	232	37.0 (0.25 to 99)	500	188	Low	AI and expert consensus (CR)	✗
Cohen et al.^37^	BoneView	N/A	N/A	N/A	637	247	Moderate	Expert consensus (CR)	✗
Dupuis et al.^38^	Rayvolve	1459	1090	8.5 (0 to 17)	2634	809	Moderate	Single expert opinion (CR)	✗
Duron et al.^11^	BoneView	242	358	57.0 (18 to 100)	600	300	Moderate	Expert consensus (CR)	✓
Gasmi et al.^43^	Rayvolve	474	404	8.3 (N/A)	878	182	Low	Expert consensus (CR)	✗
Gipson et al.^46^	Enterprise CXR TT	949	455	52 (33 to 69)	2800	134	Low	Report based (CT)	✗
Guermazi et al.^53^	BoneView	153	327	59.0 (N/A)	480	240	Moderate	Expert consensus (CR)	✓
Hayashi et al.^39^	BoneView	167	133	10.8 (2 to 21)	300	150	Moderate	Expert consensus (CR)	✓
Jacques et al.^45^	BoneView	155	141	41.1 (N/A)	296	178	Low	Expert consensus (CT)	✗
Jones et al.^40^	FractureDetect	5520	7226	> 55.0 (22 to 90)	16,019	2415	Moderate	Expert consensus (CR	✓
Nguyen et al.^44^	BoneView	167	133	10.8 (2 to 21)	300	150	Low	Expert consensus (CR)	✗
Oppenheimer et al.^41^	BoneView	309	426	61.39 (2 to 100)	1163	367	Moderate	Report based (CR)	✗
Parpaleix et al. ^42^	SmartUrgence	N/A	N/A	30.0 (16 to 52)	1772	616	Moderate	Report based (CR)	✗
Regnard et al.^35^	BoneView	N/A	N/A	N/A (1 to 103)	4774	785	High	AI & report consensus (CR)	✓
Reichert et al.^34^	Rayvolve	N/A	N/A	N/A	125	25	High	Single expert opinion (CR)	✗

*CR* conventional radiography, *CT* computed tomography, *N/A* not available.

### Risk of bias and applicability

The result of the respective RoB and applicability assessments is shown in Table 3.

**Table 3:** Tabular presentation of the QUADAS-2 results sorted by author.

Study	Risk of bias				Applicability concerns		
	Patient selection	Index test	Reference standard	Funding	Patient selection	Index test	Reference standard
Anderson et al.^36^	✓	✓	✓	✗	✓	✓	✓
Bousson et al.^50^	✓	✓	✓	✓	✓	✓	✓
Canoni-Meynet et al.^13^	✓	✓	✓	✓	✓	✓	✗
Cohen et al.^37^	✗	✓	✓	✓	?	✓	✓
Dupuis et al.^38^	✓	✓	✗	✓	✗	✓	✗
Duron et al.^11^	✓	✓	✓	✗	✓	✓	✓
Gasmi et al.^43^	✓	✓	✓	✓	✗	✓	✓
Gipson et al.^46^	✓	✓	✓	✓	✓	✓	✗
Guermazi et al.^53^	✓	✓	✓	✗	✓	✓	✓
Hayashi et al.^39^	✓	✓	✓	✗	✗	✓	✓
Jacques et al.^45^	✓	✓	✓	✓	✗	✓	✓
Jones et al.^40^	✓	✓	✓	✗	✓	✓	✓
Nguyen et al.^44^	✓	✓	✓	✓	✗	✓	✓
Oppenheimer et al.^41^	✓	✓	✗	✓	✓	✓	✗
Parpaleix et al. ^42^	✓	✓	✗	✓	✓	✓	✗
Regnard et al.^35^	✓	✓	✗	✗	✓	✓	✗
Reichert et al.^34^	✗	✓	✗	✓	?	✓	✗

✓ low risk, ✗ high risk, ? unclear risk.

Two studies^34,35^ were rated with a high RoB due an unclear reference standard, whereby one study was additionally funded by the product company and the other had an unclear patient selection. Furthermore, nine studies^11,12,36–42^ were estimated as moderate RoB studies, because one category was judged as ‘high’. Of these, five were funded^11,12,36,39,40^, three had an inadequate reference standard^38,41,42^ and one reported an inadequate patient selection^37^. A total of 6 out of 17 studies were judged to be ‘low’ in all RoB categories.

In five studies, patient selection was problematic in terms of applicability, as the selection was based exclusively on children/young adults or individual body regions^38,39,43–45^. Unclear patient selection concerning applicability were present in two studies and was already taken into consideration in the RoB rating^34,37^. There were a total of seven studies^13,34,35,38,41,42,46^ in which the methodology chosen to determine the reference standard was considered suboptimal, making their applicability inadequate. Two of these studies were categorized as “low” RoB in the overall assessment: One relied solely on CT reports^46^ and the other used a combination of expert opinion and AI^13^. A spreadsheet provides a comprehensive overview of the QUADAS-2 results in Table 4.

**Table 4:**
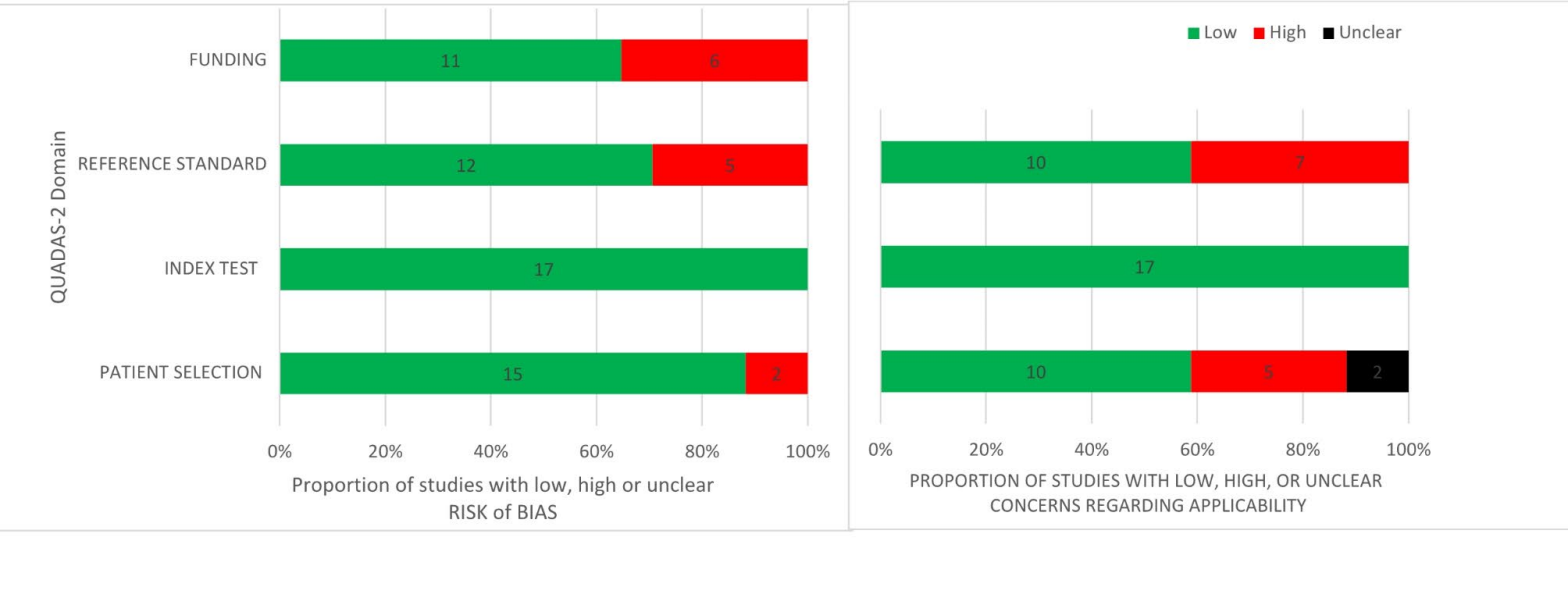
Spreadsheet presentation of the QUADAS-2 results.

### Meta-analyses

**Fig. 2:**
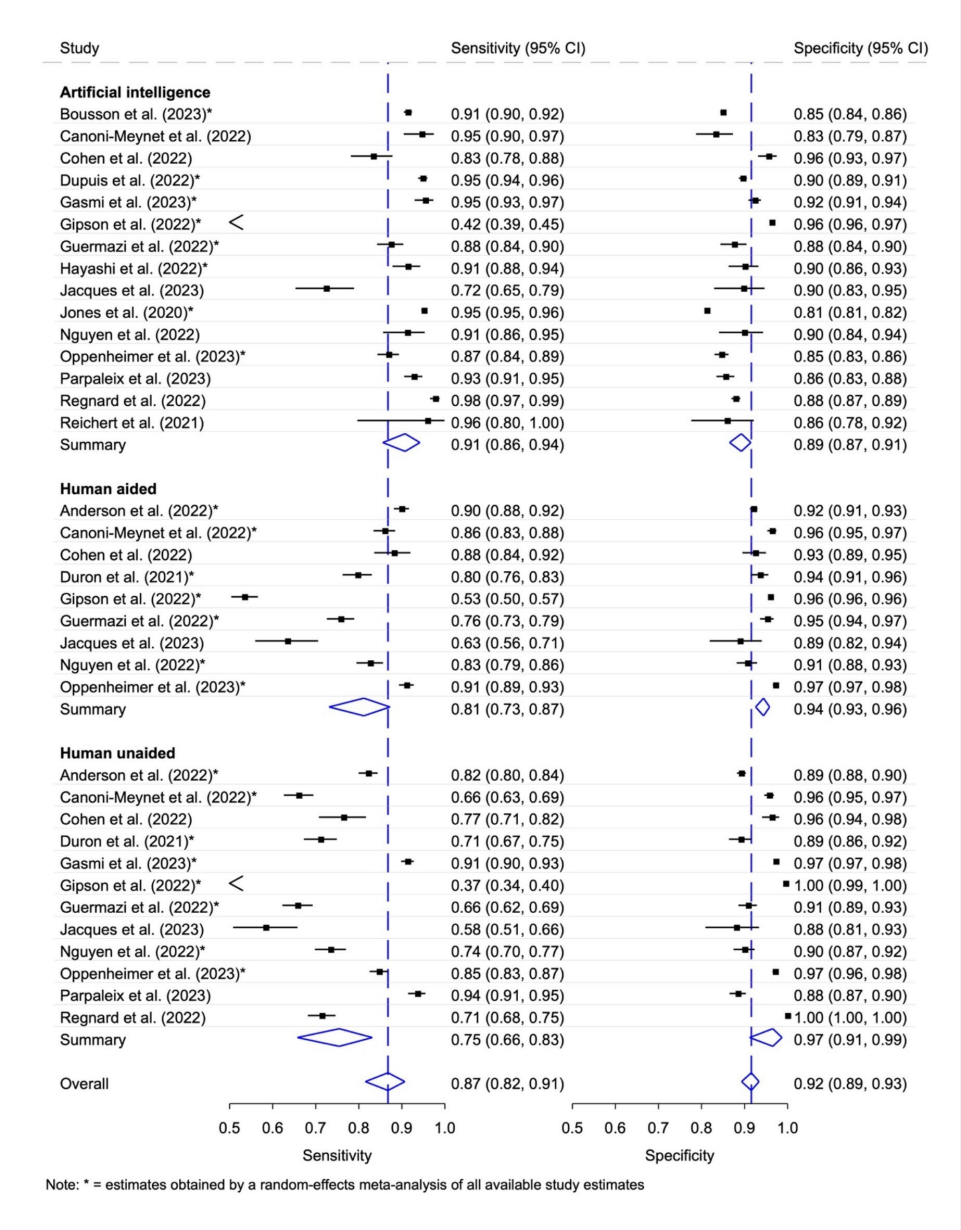
Diagnostic accuracy with 95% confidence interval (CI) according to stand-alone AI, human unaided and aided rater (total). Generalized I^2^ values: Artificial intelligence 0.86; Human aided 0.87; Human unaided 0.94; Overall 0.79.

Using sufficient information from the 17 included studies, data provided by the corresponding authors or calculations with the Wald interval error formula, 157 contingency tables were extracted for analysis. Fifteen studies presented a table of diagnostic accuracy determined using stand-alone AI, nine studies presented estimates of AI combined with human ratings, and twelve studies reported additional unaided human ratings (Fig. 2). The generalized I^2^ values for summarized heterogeneity were as followed: Artificial intelligence 0.86; Human aided 0.87; Human unaided 0.94; Overall 0.79.

### Diagnostic accuracy of stand-alone AI according to CAAI-FDS

**Fig. 3:**
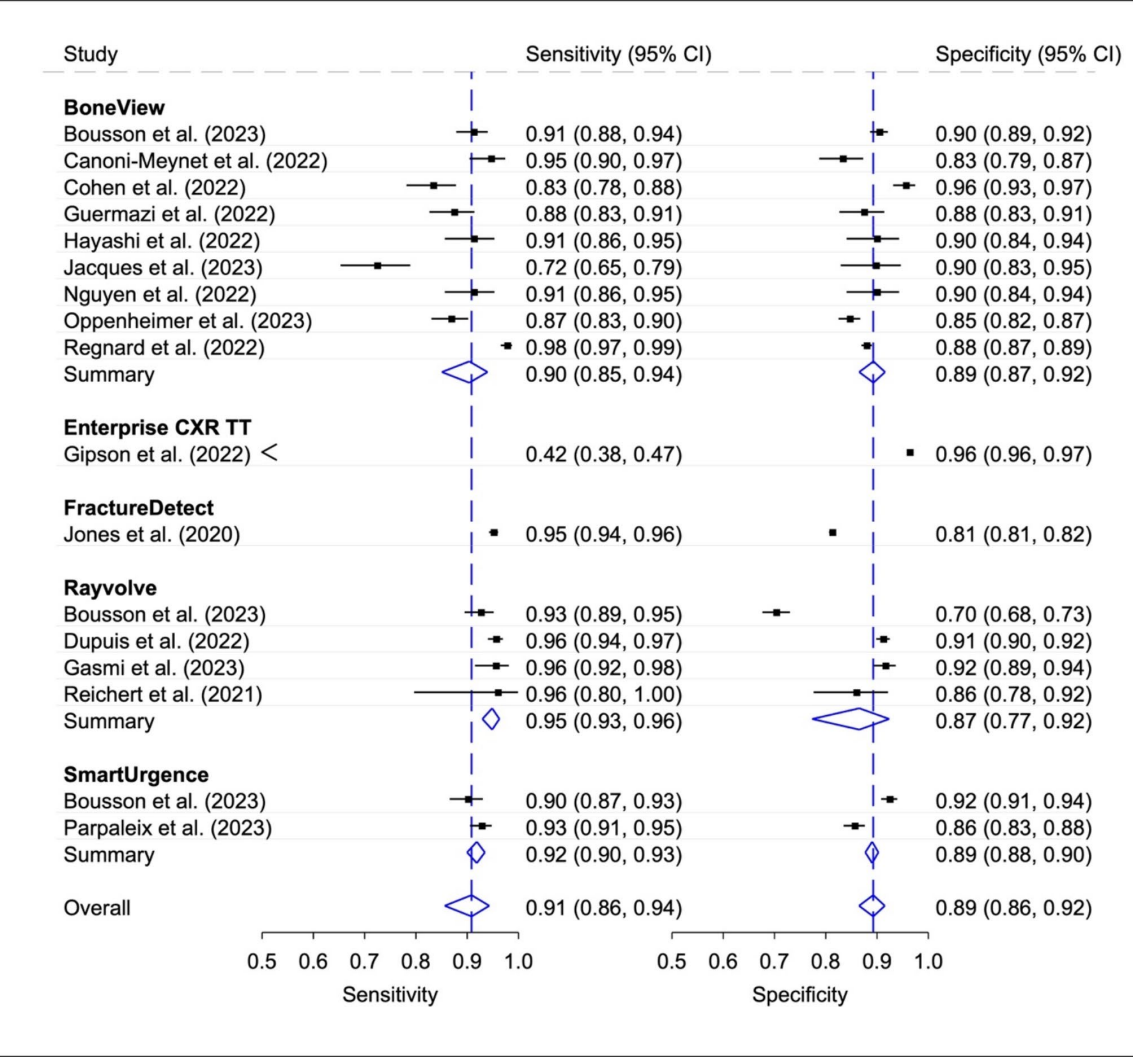
Diagnostic accuracy with 95% confidence interval (CI) according to AI fracture detection product. Generalized I^2^ values: BoneView 0.82; Enterprise CXR TT -; FractureDetect -; Rayvolve < 0.01; SmartUrgence -; Overall 0.85.

The pooled sensitivity of all stand-alone AI studies (*n* = 15) was good (0.91, 95% CI 0.86, 0.94) and the pooled specificity was moderate (0.89, 95% CI 0.87, 0.91), Fig. 3. Nine studies determined the diagnostic accuracy with *BoneView*,* four studies with Rayvolve*,* two studies with SmartUrgence*, one study each with *FractureDetect* and *Enterprise CXR Triage Trauma.* The sensitivities ranged from very poor (0.42, 95% CI 0.38, 0.47, *Enterprise CXR Trauma Triage*) to excellent (0.98, 95% CI 0.97, 0.99, *BoneView*), the specificities from poor (0.70 95% CI 0.68, 0.73, *Rayvolve*) to excellent (0.96, 95% CI 0.96, 0.97, *Enterprise CXR Trauma Triage*). Only one study compared multiple CAAI-FDS (*BoneView vs. Rayvolve vs. SmartUrgency*) with good diagnostic accuracy (sensitivity and specificity values above 0.90) for all CAAI-FDS, except for the specificity for *Rayvolve* of 0.70, 95% CI 0.68, 0.73. The generalized I^2^ values for summarized overall heterogeneity were as followed: BoneView 0.82; Enterprise CXR TT -; FractureDetect -; Rayvolve < 0.01; SmartUrgence -; Overall 0.85.

### Diagnostic accuracy of stand-alone AI according to different body regions

**Fig. 4:**
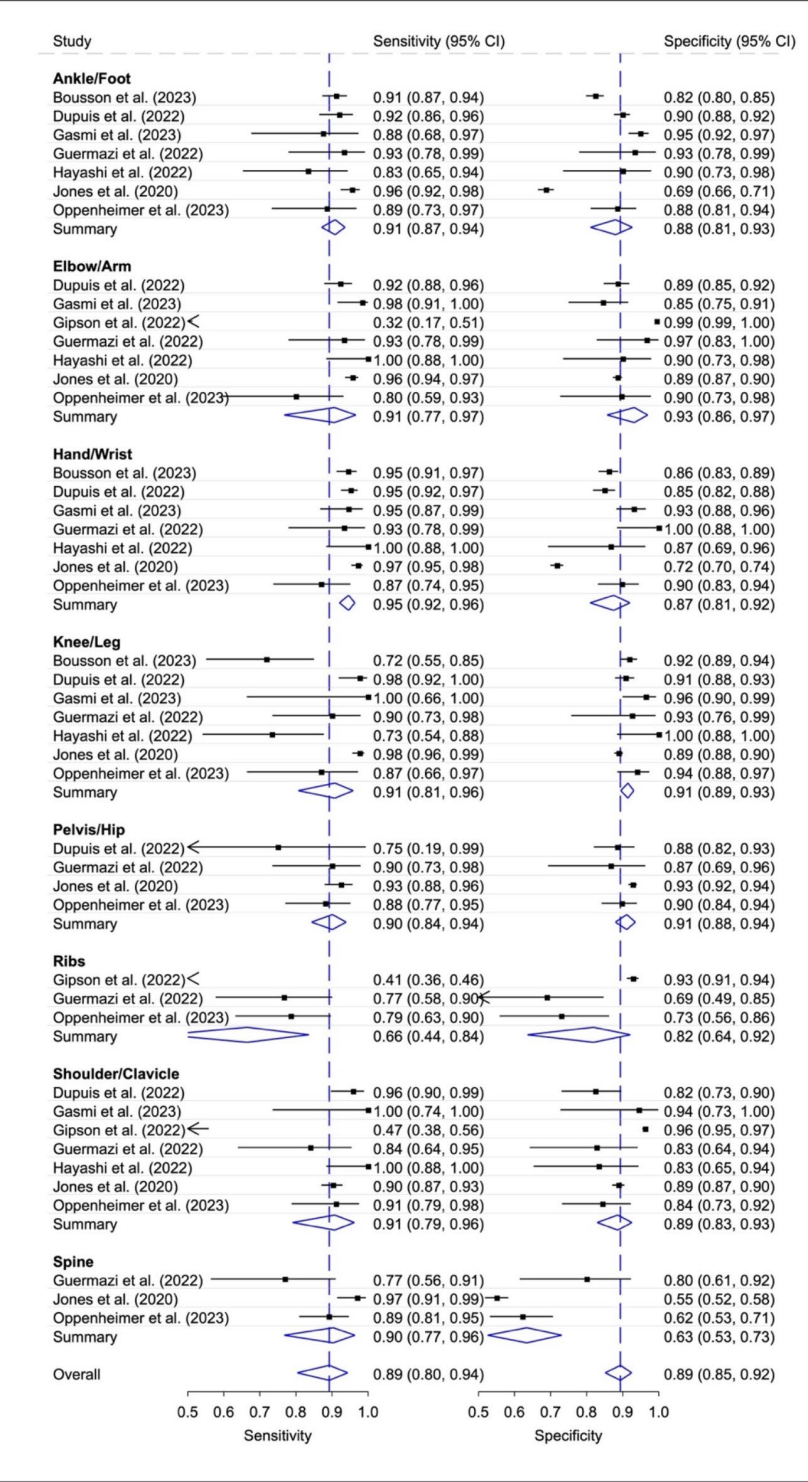
Diagnostic accuracy with 95% confidence interval (CI) for stand-alone AI according to body region. Generalized I^2^ values: Ankle/Foot < 0.01; Elbow/Arm 0.01; Hand/Wrist < 0.01; Knee/Leg 0.02; Pelvis/Hip < 0.01; Ribs < 0.01; Shoulder/Clavicle < 0.01; Spine < 0.01; Overall 0.18.

Figure 4 shows the diagnostic accuracy according to different body regions with stand-alone AI ratings. The pooled sensitivity and specificity were similar in ankle/foot, elbow/arm, knee/leg, pelvis/hip, shoulder/clavicle, and spine CR, all around 0.90. Hand/wrist showed the highest pooled sensitivity and specificity (0.95, 95% CI 0.92, 0.96 and 0.87, 95% CI 0.81, 0.92). Rib fracture detection showed in three studies the lowest pooled sensitivity (0.66, 95%: 0.44, 0.84) and specificity (0.82, 95%: 0.64, 0.92). The generalized I^2^ values for summarized overall heterogeneity were as followed: Ankle/Foot < 0.01; Elbow/Arm 0.01; Hand/Wrist < 0.01; Knee/Leg 0.02; Pelvis/Hip < 0.01; Ribs < 0.01; Shoulder/Clavicle < 0.01; Spine < 0.01; Overall 0.18. Supplement II, Supplementary Fig. 1 shows the ratings for human unaided and aided by AI for the different body regions with the corresponding generalized I^2^ values. The pooled sensitivity and specificity were higher in all body regions for the human aided ratings, except for the pooled specificity for the ribs and shoulder/clavicle.

### Diagnostic accuracy of stand-alone AI according to reference standard

**Fig. 5:**
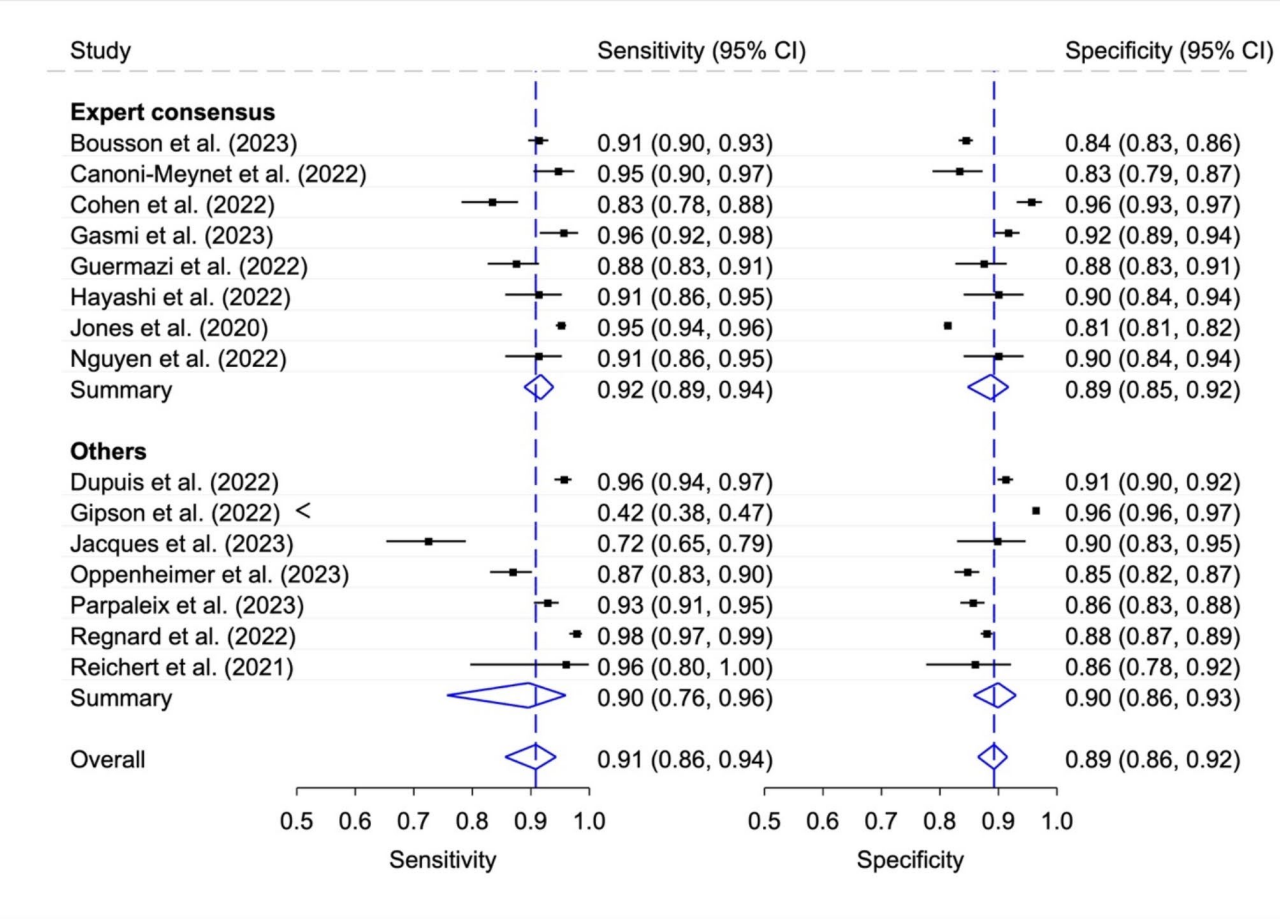
Sensitivity and specificity with 95% confidence interval (CI) for stand-alone AI according to reference standard. Generalized I^2^ values: Expert consensus 0.73; Others 0.85; Overall 0.86.

The reference standard was determined in eight studies by a group of at least two experts. The calculated pooled sensitivity was 0.92 (95% CI 0.89, 0.94) and the pooled specificity was 0.89 (95% CI 0.85, 0.92). The other seven studies determined the reference standard using other methods (see Table 2) and reported slightly different values for the pooled sensitivity and specificity (0.90, 95% CI 0.76, 0.96 and 0.90, 95% CI 0.86, 0.93) (Fig. 5). Generalized I^2^ values: Expert consensus 0.73; Others 0.85; Overall 0.86.

### Diagnostic accuracy of stand-alone AI according to industry funding status

**Fig. 6:**
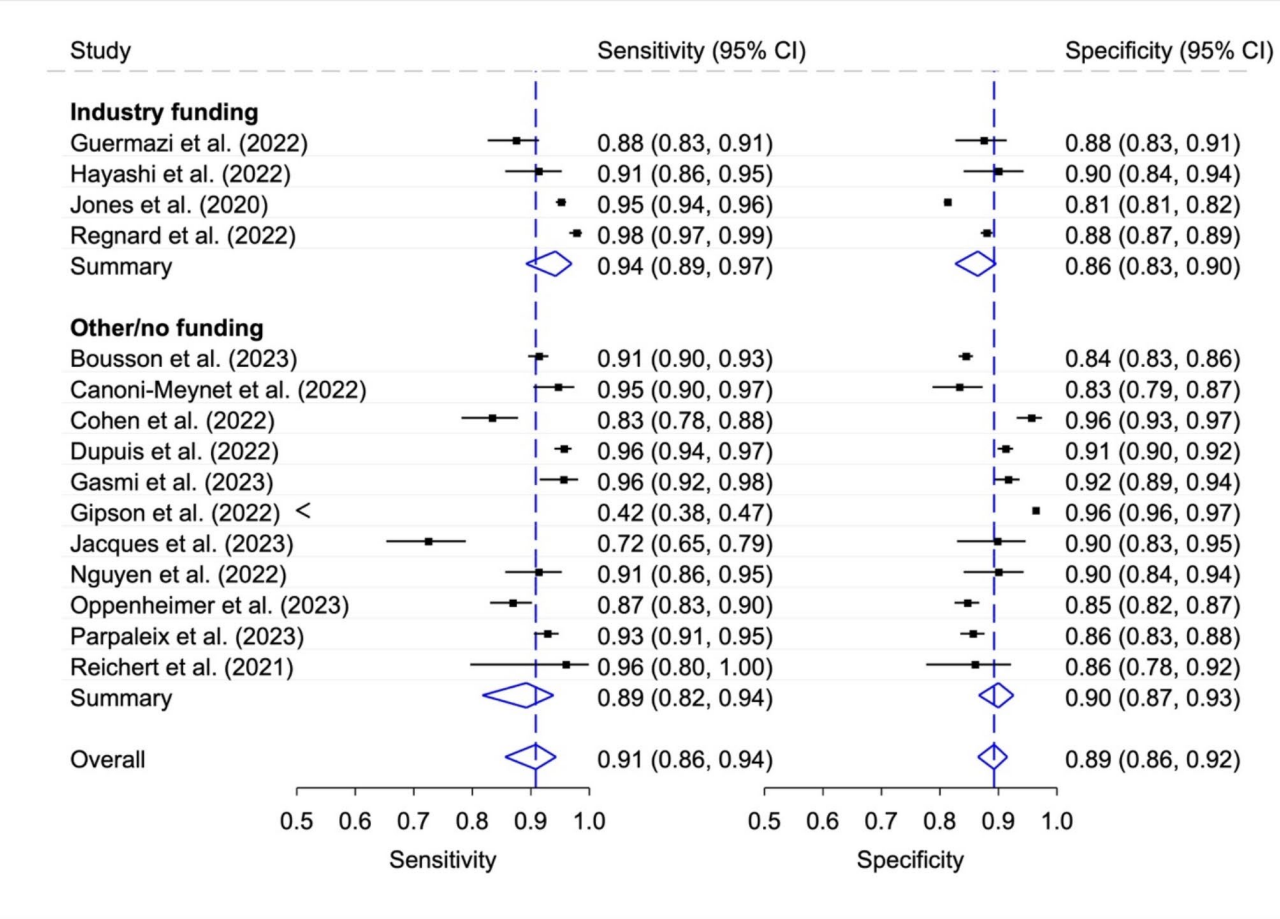
Sensitivity and specificity with 95% confidence interval (CI) for stand-alone AI according to funding status. Generalized I^2^ values: Industry funding 0.80; Other/no funding 0.86; Overall 0.86.

Four of the six funded studies reported a diagnostic accuracy of the stand-alone AI (Fig. 6). In these studies, the pooled sensitivity of 0.94 (95% CI 0.89, 0.97) was slightly higher than in the 11 non-funded studies, which had a pooled value of 0.89 (95% CI 0.82, 0.94). An opposite result was calculated for the pooled specificity (0.86, 95% CI 0.83, 0.90, with industry funding and 0.90, 95% CI 0.87, 0.93, without industry funding). The generalized I^2^ values for summarized overall heterogeneity were as followed: Industry funding 0.80; Other/no funding 0.86; Overall 0.86.

### Diagnostic accuracy of stand-alone AI according to RoB category

**Fig. 7:**
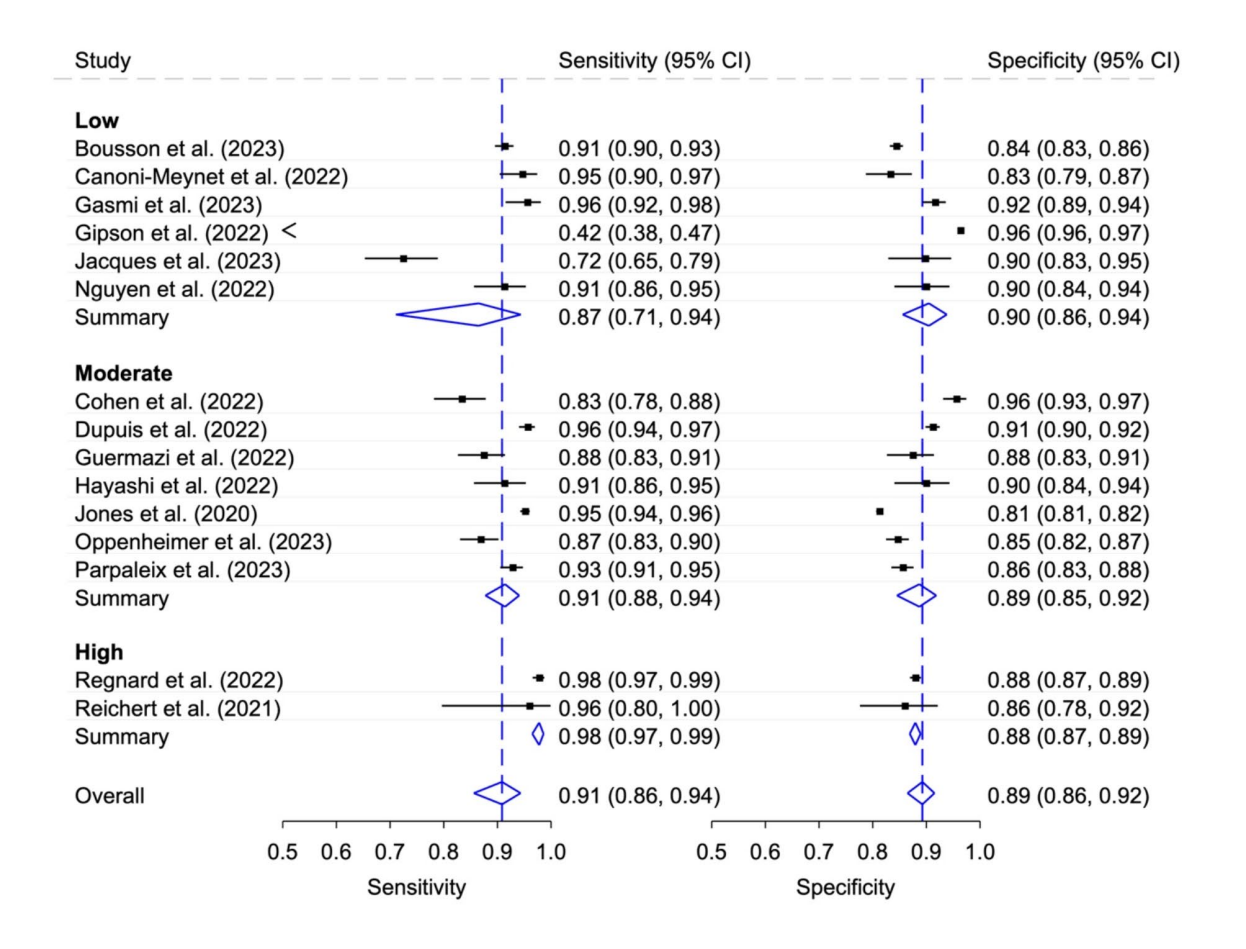
Sensitivity and specificity with 95% confidence interval (CI) for stand-alone AI according to different RoB category. Generalized I^2^ values: Low 0.87; Moderate 0.87; High -; Overall 0.86.

Six studies that were classified as low RoB studies reported the lowest pooled sensitivity but highest pooled specificity (0.87, 95% CI 0.71, 0.94 and 0.90, 95% CI 0.86, 0.94). The seven studies with moderate RoB were between the other two levels and had a pooled sensitivity of 0.91 (95% CI 0.88, 0.94) and a pooled specificity of 0.89 (95% CI 0.85, 0.92). In contrast, the two studies with high RoB showed the highest pooled sensitivity and the lowest pooled specificity (0.98, 95% CI 0.97, 0.99 and 0.88, 95% CI 0.87, 0.89). Figure 7 illustrates the corresponding forest plot. Generalized I^2^ values for summarized heterogeneity: Low 0.87; Moderate 0.87; High -; Overall 0.86.

### Diagnostic accuracy according to different rater (comparative meta-analysis)

Supplement II, Supplementary Fig. 2 shows the diagnostic accuracy in detailed rater groups with the corresponding generalized I^2^ values for summarized overall heterogeneity.

**Fig. 8:**
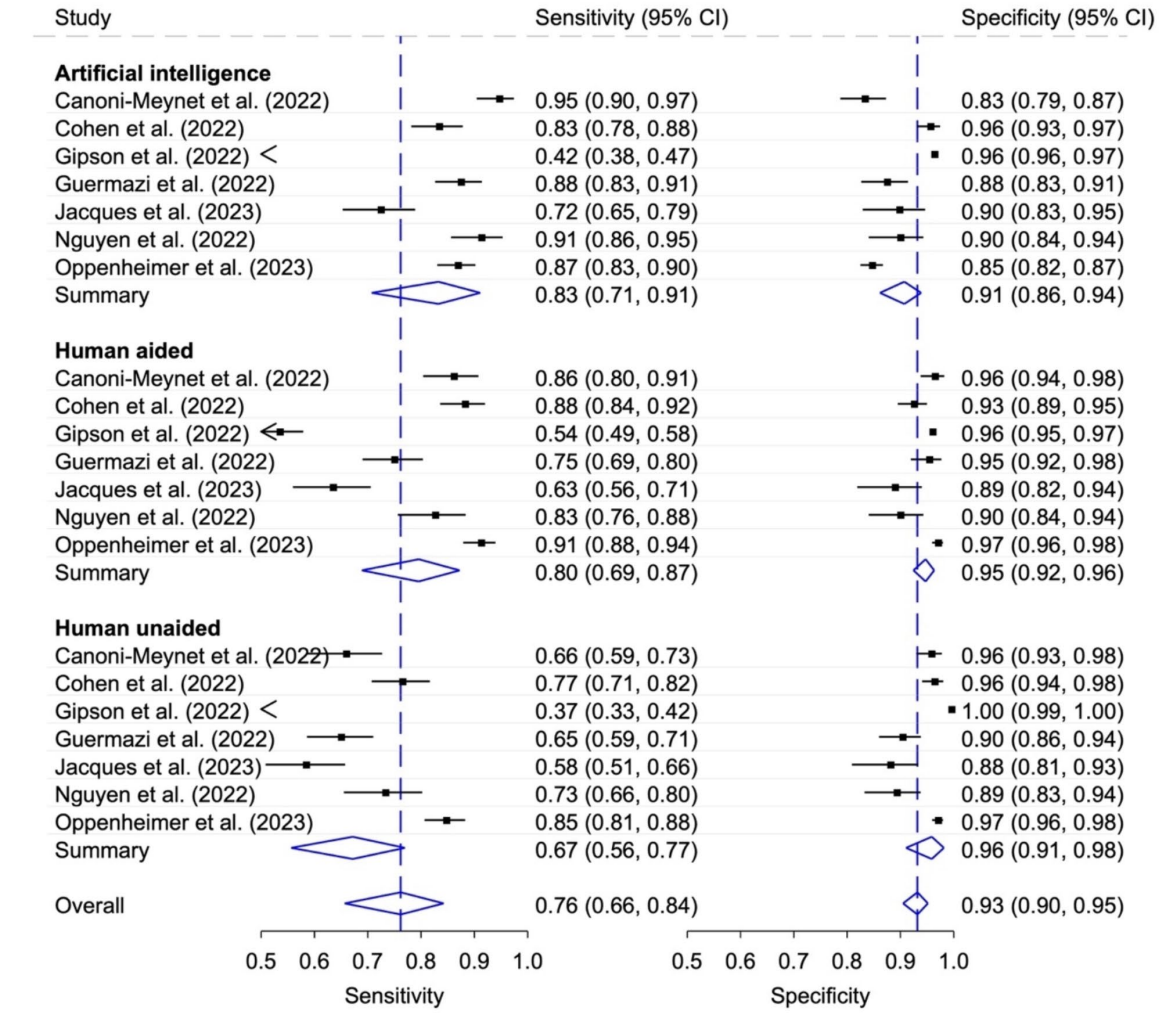
Diagnostic accuracy with 95% confidence interval (CI) depending on the type of rater (stand-alone AI and human aided/unaided).

Seven studies enabled a comparison of the accuracies between (i) stand-alone AI, (ii) human rater AI aided and (iii) human rater unaided (Fig. 8). The pooled sensitivities were for the listed rater were (i) 0.83 (95% CI 0.71, 0.91), (ii) 0.80 (95% CI 0.69, 0.87), (iii) 0.67 (0.56, 0.77) and for the pooled specificities (i) 0.91 (95% CI 0.86, 0.94), (ii) 0.95 (95% CI 0.92, 0.96), and (iii) 0.96 (95% CI 0.91, 0.98), respectively. The generalized I^2^ values for summarized overall heterogeneity were as followed: Artificial intelligence 0.86; Human aided 0.86; Human unaided 0.91; Overall 0.67. Table 5 shows the relative sensitivity and specificities of the comparative meta-analysis. When restricting the analysis to independent, non-industry funded, studies (see Supplementary Fig. 3 with generalized I^2^ values), the sensitivity did not differ significantly between stand-alone AI and human rater AI aided (*p* = 0.316) and the specificity was significantly higher in human AI rater aided (*p* < 0.001). The sensitivity was significantly smaller in human rater unaided studies compared to stand-alone AI and human rater AI aided or (both *p* ≤ 0.001) and the specificity did not differ significantly between the two human rater groups (*p* = 0.316).

**Table 5:** Relative sensitivity and specificity with 95% confidence interval (95% CI) for pairwise comparisons of comparative meta-analysis of studies comparing Artificial intelligence (stand-alone) vs. human unaided vs. human aided in all studies and in unfunded studies only.

	All studies (*n* = 6*)		Unfunded studies only (*n* = 5^$^)	
	Relative measure (95% CI)	*p*-value	Relative measure (95% CI)	*p*-value
AI vs. human unaided				
Relative sensitivity	1.19 (1.13, 1.26)	*p* < 0.001	1.16 (1.10, 1.24)	*p* < 0.001
Relative specificity	0.91 (0.89, 0.94)	*p* < 0.001	0.91 (0.88, 0.94)	*p* < 0.001
Human aided vs. unaided				
Relative sensitivity	1.15 (1.08, 1.22)	*p* < 0.001	1.15 (1.08, 1.23)	*p* < 0.001
Relative specificity	1.00 (0.98, 1.02)	*p* = 0.945	0.99 (0.98, 1.01)	*p* = 0.422
AI vs. human aided				
Relative sensitivity	1.04 (1.01, 1.08)	*p* = 0.021	1.02 (0.98, 1.05)	*p* = 0.316
Relative specificity	0.92 (0.90, 0.94)	*p* < 0.001	0.91 (0.89, 0.94)	*p* < 0.001

*See, Fig. 8; ^$^ see supplementary Fig. 3.

## Discussion

This meta-analysis compared 17 studies using CAAI-FDS based on CR and found good diagnostic accuracy across most tested AI tools and anatomical regions except for ribs and spine, with the highest performance achieved when used in conjunction with human assessment. The impact of industry funding of the studies on diagnostic accuracy was close low.

In our CAAI-FDS analysis, the BoneView tool was the most frequently studied and showed a pooled sensitivity of 0.90 (95% CI 0.85, 0.94) and specificity of 0.89 (95% CI 0.87, 0.92). The second most frequently investigated product with only four studies, Rayvolve, had a higher pooled sensitivity of 0.95 (95% CI 0.93, 0.96), but at the same time a slightly lower specificity of 0.87 (95% CI 0.77, 0.92). Of these four studies, two were exclusively conducted for pediatric fractures^38,43^, and one study^34^ was judged to be highly biased with the smallest sample size of only 125 within the entire meta-analysis, which made the evaluation of the diagnostic performance of Rayvolve challenging. The same applied to the Enterprise CXR Triage Trauma (TT), FractureDetect and SmartUrgence products due to a lack of publications.

The performance of stand-alone AI showed overall comparable results for the different body regions, except for the ribs, which had a pooled sensitivity of 0.66 (95% CI 0.44, 0.84), and the spine, reporting a pooled specificity of 0.63 (95% CI 0.53, 0.73). The lower sensitivity in the assessment of rib fractures suggested that a stand-alone AI may have difficulty detecting minor or subtle fractures superimposed on CRs by other bony structures, whereas experimental algorithms already had higher values of sensitivity around 0.90 ^47,48^. Nevertheless, even humans without AI assistance had considerable difficulty in detecting rib fractures on CR^41,46^. In contrast, our results suggested that AI appeared to frequently interpret false-positive results for spinal fractures. This assumption was contradicted by Rosenberg et al. (2022)^49^, who reported a specificity of over 80% in the detection of thoracolumbar fractures using two different non-commercially available deep learning networks. Taking these results into account, it appeared that the ribs and spine in particular require more extensive training sets and validation testing for the CAAI-FDS, which is why AI in fracture detection, especially in these regions of the body, cannot yet be considered a definitive solution in countries with limited access to CT scanners^50^.

Our results indicated that stand-alone AI could detect fractures with high accuracy. At the same time, there was a tendency to detect non-existent fractures, which was much less common in human raters without AI support (pooled specificity: 0.96, 95% CI 0.91, 0.98). In general, clinicians who were less involved in the daily assessment of radiographs or had less experience in diagnosis seemed to benefit particularly from the use of AI. The pooled sensitivity improved from 0.67 (95% CI 0.56, 0.77) to 0.80 (95% CI 0.69, 0.87) with the support of a CAAI-FDS in the present study. Neither the demographic characteristics of the study participants, the experience of the raters nor the location of the fracture influenced the improvement of diagnostic accuracy using AI support^12,13,44^.

This study shows that industry funding is associated with a 5% improvement in sensitivity and a 3% reduction in specificity. Vendor sponsored publications may have introduced bias through selective reporting and optimized study designs that favored positive results, such as participant selection and definition of endpoints that emphasized the strengths of CAAI-FDS. These practices may have led to an overestimation of the effectiveness of CAAI-FDS. Although industry funding can drive innovation and provide the necessary resources for research, a critical assessment of potential conflicts of interest was also required, so independent validation of these results was necessary to ensure their robustness and minimize bias. Emphasizing transparency in reporting and the inclusion of non-sponsored studies would contribute to a more balanced and accurate understanding of the diagnostic performance of CAAI-FDS. In addition, studies should explicitly disclose funding sources and potential conflicts of interest to maintain the integrity of the research and confidence in the results.

The studies did not reveal substantial differences in the diagnostic accuracy between the various reference standards, as the pooled values for sensitivity and specificity were around 0.90. Almost half of the studies were using a reference standard created by a group of experts, some of whom were very heterogeneous. Despite this consistency, two outliers could be identified that used either CT reports or CT images as a reference standard. The study by Gipson et al.^46^ reported the lowest sensitivity (0.42, 95% CI 0.38, 0.47), followed by the study by Jacques et al.^45^ (0.72, 95% CI 0.65, 0.79). Jacques et al. used CT images as ground truth and highlighted that studies using CR as the reference may underestimate the number of missed fractures and that their results might be closer to clinical reality^45^. This study was limited to detecting hand and wrist fractures, but comparable studies using CR images as a reference reported a higher sensitivity^33,37^.

Compared to three other meta-analyses^14,15,51^, our results are close to the other studies. In the overall analysis of all included studies, the stand-alone AI showed a sensitivity of 0.91 (95% CI 0.86, 0.94), but specificity of 0.89 (95% CI 0.87, 0.91). Yang et al.^14^ reported a pooled sensitivity of 87% (95% CI 78, 93) and a specificity of 91% (95% CI 85, 95) in their meta-analysis of nine studies. In contrast to our research, the deep learning protocols used in the presented studies were not commercially available and thus were not included in our meta-analysis.

In a more recent meta-analysis Kuo et al.^15^ included 32 studies and reported a pooled sensitivity of 91% (95% CI 84, 85) and a specificity of 91% (95% CI 81, 95) for stand-alone AI which was comparable to human unaided judgment. In agreement to our findings, the authors reported an improved clinician performance when AI was used in addition to human judgment. They also highlighted the problem of underestimating clinician performance since most studies provided clinicians with no background clinical information. With one exception^11^, the included studies differed from our meta-analysis, as mostly experimental algorithms were investigated.

The third and final meta-analysis by Zhang et al.^51^ also revealed a similar picture. Of the 39 analyzed studies, only one study^34^ was included in our meta-analysis, as commercial availability was again not of interest. The pooled sensitivity and specificity of AI alone were 0.90 (95% CI 0.87–0.92) and 0.92 (95% CI 0.90–0.94), respectively, indicating slightly better specificity compared to our results. Surprisingly, the results from multicenter studies showed higher sensitivity (0.92 vs. 0.88) and specificity (0.94 vs. 0.91) compared to the results from single-center studies^51^.

Recently, a comprehensive review of CAAI-FDS on CR and CT images was published to provide information and evidence to assist healthcare facilities in decision making and product implementation^52^. The authors presented 21 CAAI-FDS from 15 different AI vendors, 14 of which are intended for fracture detection on CR. In comparison to this work, the additionally identified studies were not meta-analyzed.

The overall impact of CAAI-FDS is already remarkable. Studies reported that the time required for interpretation of each patient case for fracture detection can be reduced between 6.3 and 11.6 s with the aid of AI^11–13^. This reduction, when spread over a large number of cases, can decrease the overall workload of radiologists significantly to focus on more complex cases. However, the solutions may not only save time, but also detect concomitant injuries such as joint effusions, dislocations or bone lesions that clinicians might not have recognized^35^. Furthermore, as mentioned above, the pooled sensitivity using CAAI-FDS improved from 0.67 to 0.80. This improvement may lead to better patient outcomes through more accurate and timely diagnoses. In addition, AI-assisted tools in musculoskeletal imaging impact the clinical workflow and specifically support the decision-making process in imaging prescription, report writing, image acquisition time, image interpretation and final report dictation to reduce workload^53^. Accordingly, a harmonized application of different AI solutions may lead to relieve the burden on clinicians and contribute to a reduction in overcrowded ED. In terms of cost-effectiveness, no significant initial investment is expected for the implementation of AI systems, as the software can simply be installed on existing hardware and integrated into the Picture Archiving and Communication System (PACS) or the Radiological Information System (RIS). Product prices vary depending on the vendor and are often based on the number of users, installations, and analyses performed. Conversely, AI can help reduce costs associated with misdiagnosis, including additional tests, treatments, potential legal liabilities, and more efficient use of clinicians’ time. In general, better diagnostic accuracy can lead to faster and more effective treatments, reducing overall healthcare costs. However, possible barriers to implementation include the general costs, which could be a challenge for low-income countries. Regular training of staff in the use of CAAI-FDS and resistance to new changes may also be problematic. Additionally, regulatory approvals for new AI solutions can be lengthy and complex, significantly delaying their integration into clinical practice.

The present study has several limitations. A key limitation is that the DIAG and ACR DSI AI Central databases were used for the study. This choice was based on the transparent and good overview of available AI solutions, but it remains unclear how regularly these databases are updated and how complete they are. In addition, the heterogeneity of the included studies is due to differences in study design, demographic characteristics and body regions examined, which can lead to inconsistencies that make direct comparison difficult. For example, the resolution of the imaging techniques used in the studies is often unknown and may vary, which can affect diagnostic accuracy. Moreover, studies that have focused on either pediatric or adult patients may have different measures of diagnostic accuracy due to fractures and bone densities for the respective age groups. Furthermore, studies targeting different anatomical regions can be diagnostic challenging. For instance, detecting fractures in the wrist region often involves differentiating between various types of fractures, such as radius and carpal bones fractures, each requiring specific diagnostic criteria. Differences in CAAI-FDS implementation across studies, including how they are trained, validated and deployed, further contribute to this heterogeneity. These differences could affect the accuracy of the pooled results and overestimate their applicability on clinical practice. Another critical limitation is the possible bias caused by industry funded trials. Sponsored studies could exhibit selective reporting that focuses on positive and omits negative results, creating an exaggerated picture of the effectiveness of CAAI-FDS. For example, a sponsored study might emphasize the high sensitivity of the CAAI-FDS, while not adequately reporting cases of false positives or cases where the AI did not detect fractures. This selective reporting may bias the meta-analysis towards more positive results and compromise the overall objectivity. In addition, only patient-related sensitivity and specificity were considered in this meta-analysis, as an analysis of fracture-related values was not possible for all studies due to incompleteness. To maintain methodological and analytical homogeneity, CAAI-FDS studies based on CT imaging were excluded. Lastly, this systematic review was not pre-registered.

Giving these limitations, future research is necessary. Despite the promising products, their application possibilities and diagnostic accuracy, further detailed studies are required, as the products OsteoDetect and RBfracture, for example, have so far only been examined by the developers. Also, the differences in study design, test set demographics and results strongly emphasize the need for further research to better understand the performance of the different products and evaluate their applicability in clinical practice. Real-world scenarios in different clinical settings are required to test and validate the performance of the CAAI-FDS. Developing and adhering to standardized protocols for study methods and presentation of results can reduce heterogeneity and improve the comparability of results of DTA studies in the field of AI solutions. In addition, larger and independent studies on the individual regions of the body are required to localize possible weaknesses that have already been identified in our results. Furthermore, future studies are recommended to use CT as a gold standard. Yang et al.^14^ have already recommended to define a clear reference standard. A plausible definition might be that conventional radiographs should be from patients who have undergone surgical internal fixation of the fracture. Radiographs without expected fractures should be confirmed by expert consensus based on CT imaging. Lastly, as initial studies showed promising results for the diagnostic accuracy of rib and spine fracture detection on CT images^54–56^, but these were excluded for methodological and analytical reasons in our study, further meta-analyses on fracture detection using AI on CT images are required, including the investigation of proof-of-concept studies. In the future, initial studies should also examine the potential economic benefits of CAAI-FDS to provide hospital providers with further incentives to implement these AI solutions. Finally, transparent reporting of AI studies, including clear disclosure of funding sources and potential conflicts of interest, is needed. Further non-sponsored research should be conducted to validate the results and reduce the impact of potential bias in the future.

The introduction of CAAI-FDS has the potential to significantly improve healthcare systems by increasing efficiency and diagnostic accuracy. Automated analysis of CR could reduce the burden on clinical staff, allowing them to focus on more complex cases and reduce turnaround times, which can be critical in emergency situations. Consistent AI interpretation could reduce human error and enable early detection and timely intervention, which could improve patient outcomes. In addition, CAAI-FDS could optimize resource allocation and reduce costs associated with misdiagnosis and unnecessary testing. The integration of these systems could also improve access to quality care in underserved areas by providing reliable diagnostic support.

## Conclusions

This meta-analysis provides a comprehensive assessment of the diagnostic accuracy of CAAI-FDS on CR images by synthesizing data from multiple studies. Several key insights and actionable recommendations emerge from our study, which can inform decision-making for clinicians and policymakers.

The findings indicate that implementing CAAI-FDS can achieve the best diagnostic accuracy when AI is combined with human assessments, providing a valuable second opinion that can enhance diagnostic confidence and accuracy. Clinicians should be aware that AI demonstrates higher diagnostic accuracy in certain anatomical regions such as the wrist and ankle. Conversely, extra caution should be taken when using AI for diagnosing spine and rib fractures, where AI accuracy is comparatively lower. Additionally, clinicians should stay updated with the latest AI developments and understand the strengths and limitations of the AI systems they use.

Implementing feedback channels to report AI performance issues or discrepancies will help vendors improve their CAAI-FDS and ensure they meet clinical requirements. Developing standards for CAAI-FDS implementation in clinical settings, including guidelines for training, validation, and continuous monitoring of AI solutions, is essential to ensure reliable performance. Independent research studies that validate the products are crucial for providing unbiased information about their capabilities and limitations. Implementing regulations requiring full disclosure of funding sources and potential conflicts of interest in AI research publications is vital to maintain trust in AI solutions. Using high-resolution CT imaging as a reference standard in validating CAAI-FDS will enhance their reliability and clinical utility. Furthermore, the creation of centralized databases that collect and share anonymized imaging data from diverse populations can improve AI training datasets and contribute to the development of improved CAAI-FDS.

Simultaneously, greater market transparency is critical to enable healthcare organizations to make informed decisions about adoption. Policy makers should consider developing comprehensive guidelines and policies that support the integration of CAAI-FDS into clinical practice and ensure equitable access to these technologies across healthcare organizations. This includes removing potential barriers to adoption to maximize the benefits of AI in improving diagnostic accuracy and healthcare efficiency.

## Electronic supplementary material

Below is the link to the electronic supplementary material.

Supplementary Material 1

Supplementary Material 2

## Data Availability

The data that support the findings of this study are available from the corresponding author upon reasonable request.
