# Supplementary material for: Artificial intelligence in commercial fracture detection products: a systematic review and meta-analysis of diagnostic test accuracy
Source: Sci Rep. 2024 Oct 4;14:23053. doi: 10.1038/s41598-024-73058-8 (PMC11452402; doi:10.1038/s41598-024-73058-8)
Supplement: Supplementary file 1 — Supplementary Material 1 [file 41598_2024_73058_MOESM1_ESM.docx]

# Artificial Intelligence in Commercial Fracture Detection Products: A Systematic Review and Meta-Analysis of Diagnostic Test Accuracy – Supplementary document

## ***Analytic code (Do-Files) for STATA 18.1 (StataCorp, College Station, TX, USA)***

### **MAIN analytic code – Filename: MAIN.do**

// Stata 18

ssc install metadta

ssc install resize

*********************************************

******* CLEAR & LOAD HELP PROGRAMS *****

*********************************************

// To run this Do-File : in the working directory the following three items are needed: 1. raw_dataset.dta, 2. CLEAN_DATASET.do, 3. HELPPROGRAMS.do

clear all

cls

do "HELPPROGRAMS.do"

*********************************************

*********. LOAD & CLEAN DATASET *******

*********************************************

use raw_dataset.dta, clear

do CLEAN_DATASET.do

*********************************************

******** ANALYSIS ********

*********************************************

******* Figure_2

by_aggr_group_forest, var(study_id) byvar(ai_human_combined) pdfname(Figure_2) title("") model(random) color(blue) save_dta(0) size(3) software(0) note(Note: * = estimates obtained by a random-effects meta-analysis of all available study estimates, size(*1))

******* Figure_3-7, Suppl_Figure_1-2

drop by_group_reader_4567 by_group_reader_123total

foreach var of varlist by_group_* {

if "`var'" == "by_group_ai" local pdf_name Figure_3

if "`var'" == "by_group_bodyregion" local pdf_name Figure_4

if "`var'" == "by_group_reference" local pdf_name Figure_5

if "`var'" == "by_group_funding" local pdf_name Figure_6

if "`var'" == "by_group_risk" local pdf_name Figure_7

if "`var'" == "by_group_reader_123" local pdf_name Figure_8

if "`var'" == "by_group_bodyregionunaided" local pdf_name Suppl_Figure_1a

if "`var'" == "by_group_bodyregionaided" local pdf_name Suppl_Figure_1b

if "`var'" == "by_group_reader_total" local pdf_name Suppl_Figure_2

preserve

keep if !missing(`var')

local lbe: variable label `var'

local by_var = lower("`lbe'")

bygroup_forest, var(`by_var') model(random) color(blue) save_dta(0) size(3.5) pdfname(`pdf_name')

restore

}

******* SUPPL_Figure_3

clonevar by_group_reader_123_nofund = by_group_reader_123

label var by_group_reader_123_nofund "Reader"

replace by_group_reader_123_nofund = . if funding == 1

preserve

keep if !missing(by_group_reader_123_nofund)

bygroup_forest, var(reader) model(random) color(blue) save_dta(0) size(3.5) pdfname(Suppl_Figure_3)

restore

*************************************************

*********. AGGREGATED & COMPARATIVE ********

*************************************************

******* Table_5

*** i=0 => all studies; i = 1 => without funding

forval i = 0/1 {

*** AI vs. Unaided.

preserve

keep if !missing(by_group_reader_123) `funding_condition'

drop if by_group_reader_123 == 3

gen ai = reader == 1

label define ref_ai_combined 1 "Artificial intelligence" 0 "Human unaided"

label values ai ref_ai_combined

by_aggr_group_forest_comp, var(study_id) byvar(ai) pdfname(Table_5a`i') title("") model(random) color(blue) save_dta(0) size(3.4) software(0) note("{bf:Note:} * = estimates obtained by a random-effects meta-analysis of all available study estimates") noteopt(size(*1.2))

restore

*** Aided vs. Unaided

preserve

keep if !missing(by_group_reader_123) `funding_condition'

drop if by_group_reader_123 == 1

gen ai = reader == 3

label define ref_ai_combined 1 "Human aided" 0 "Human unaided"

label values ai ref_ai_combined

by_aggr_group_forest_comp, var(study_id) byvar(ai) pdfname(Table_5b`i') title("") model(random) color(blue) save_dta(0) size(3.4) software(0) note("{bf:Note:} * = estimates obtained by a random-effects meta-analysis of all available study estimates") noteopt(size(*1.2))

restore

*** Aided vs. AI

preserve

keep if !missing(by_group_reader_123) `funding_condition'

drop if by_group_reader_123 == 2

gen ai = reader == 3

label define ref_ai_combined 1 "Human aided" 0 "Artificial intelligence"

label values ai ref_ai_combined

by_aggr_group_forest_comp, var(study_id) byvar(ai) pdfname(Table_5c`i') title("") model(random) color(blue) save_dta(0) size(3.4) software(0) note("{bf:Note:} * = estimates obtained by a random-effects meta-analysis of all available study estimates") noteopt(size(*1.2))

restore

local funding_condition = "& funding == 0"

}

### **CLEAN DATASET analytic code – Filename: CLEAN_DATASET.do**

drop if missing(Study_id)

capture gen author = First_author + " et al. (" + string(Year)+")"

label var author "Study"

rename _all, lower

**** From % to proportion for sens & spec

qui foreach vor in sens spec{

replace `vor'_es = `vor'_es/100

replace `vor'_lci = `vor'_lci/100

replace `vor'_uci = `vor'_uci/100

}

*** Calculate tp, fn, tn, fp out of sens and CI if possible

gen tp_fn_calculation_possible = (missing(tp) | missing(fn)) & !missing(sens_es) & !missing(sens_lci)

gen tn_fp_calculation_possible = (missing(tn) | missing(fp)) & !missing(spec_es) & !missing(spec_lci)

replace tp = round(0.5 * ((sens_es * (sens_es*(1-sens_es)) / ((sens_es-sens_lci)/1.96)^2)) + 0.5 * ((sens_es * (sens_es*(1-sens_es)) / ((sens_uci-sens_es)/1.96)^2))) if tp_fn_calculation_possible == 1

replace fn = round(0.5*(1-sens_es)*(sens_es*(1-sens_es)) / ((sens_es-sens_lci)/1.96)^2 + 0.5*(1-sens_es) * (sens_es*(1-sens_es)) / ((sens_uci-sens_es)/1.96)^2) if tp_fn_calculation_possible == 1

replace tn = round(0.5 * ((spec_es * (spec_es*(1-spec_es)) / ((spec_es-spec_lci)/1.96)^2) + (spec_es * (spec_es*(1-spec_es)) / ((spec_uci-spec_es)/1.96)^2))) if tp_fn_calculation_possible == 1

replace fp = round(0.5*(1-spec_es)*(spec_es*(1-spec_es)) / ((spec_es-spec_lci)/1.96)^2 + 0.5*(1-spec_es) * (spec_es*(1-spec_es)) / ((spec_uci-spec_es)/1.96)^2) if tp_fn_calculation_possible == 1

*** CHECK DEVIATION FROM PRESENT DATA

gen study_id_dummy = "Estimate " + string(no_uniquie)

isid study_id_dummy

cls

metadta tp fp fn tn, nofplot nosroc study(study_id_dummy) download(all_studies)

preserve

use all_studies, clear

keep _ES _LCI _UCI _PARAMETER _LABEL

rename _LABEL study_id_dummy

reshape wide _ES _LCI _UCI, i(study_id_dummy) j(_PARAMETER)

rename (_ES0 _LCI0 _UCI0) (spec_es_m spec_lci_m spec_uci_m)

rename (_ES1 _LCI1 _UCI1) (sens_es_m sens_lci_m sens_uci_m)

drop if missing(study_id_dummy) | study_id_dummy=="Overall"

save all_studies_diag_accuracy, replace

restore

merge 1:1 study_id_dummy using all_studies_diag_accuracy

drop _merge

local deviation_allowed = 0.01

gen unplausible = ////

abs(sens_es-sens_es_m)>`deviation_allowed' | abs(sens_lci-sens_lci_m)>`deviation_allowed' | abs(sens_uci-sens_uci_m)>`deviation_allowed' | ////

abs(spec_es-spec_es_m)>`deviation_allowed' | abs(spec_lci-spec_lci_m)>`deviation_allowed' | abs(spec_uci-spec_uci_m)>`deviation_allowed' ////

if !missing(sens_es) & !missing(sens_lci) & !missing(sens_uci) & ////

!missing(sens_es_m) & !missing(sens_lci_m) & !missing(sens_uci_m) & ////

!missing(spec_es) & !missing(spec_lci) & !missing(spec_uci) & ////

!missing(spec_es_m) & !missing(spec_lci_m) & !missing(spec_uci_m)

drop if unplausible == 1 & tp_fn_calculation_possible == 1

*** Cleaning

label var tp "TP"

label var tn "TN"

label var fp "FP"

label var fn "FN"

foreach var2 in sens spec {

foreach what in es lci uci {

tostring `var2'_`what', gen(`var2'i_`what') format(%3.2f) force

}

gen `var2'it_95ci = `var2'i_es + " [" + `var2'i_lci + ", " + `var2'i_uci + "]"

}

label var sensit_95ci "Sensitivity (95% CI)"

label var specit_95ci "Specificity (95% CI)"

clonevar dummy = reader

gen ai_human_combined = 1 if dummy == 1

replace ai_human_combined = 3 if dummy == 4 | dummy == 5 | dummy == 2

replace ai_human_combined = 2 if dummy == 3 | dummy == 6 | dummy == 7

drop dummy

label define rater_num_lab 1 "Artificial intelligence" 2 "Human aided" 3 "Human unaided"

label values ai_human_combined rater_num_lab

label var ai_human_combined "Type of rating"

drop tp_fn_calculation_possible tn_fp_calculation_possible study_id_dummy spec_es_m spec_lci_m spec_uci_m sens_es_m sens_lci_m sens_uci_m unplausible

drop by_group_younger21 by_group_older21

drop if missing(tp) | missing(fn) | missing(tn) | missing(fp)

drop if (fn == 0 & tp == 0) | (fp == 0 & tn == 0)

rm all_studies_diag_accuracy.dta

rm all_studies.dta

### **HELP PROGRAMS analytic code – Filename: HELPPROGRAMS.do**

*** PROG CREATES A DTA-FORESTPLOT USING METADTA BY A SPECIFIED SUBGROUP AND SAVES IT TO PDF

capture prog drop bygroup_forest

program bygroup_forest

syntax, var(name) [model(string) pdfname(string) color(string) save_dta(integer 0) size(real 2) software(integer 0) note(string) title(string)]

version 18.0

qui{

preserve

if "`pdfname'" == "" local pdfname = `var'_forrest

if `save_dta' == 1 local download download(dta_`var')

keep if !missing(`var')

capture drop ai_software_string

decode ai_software, gen(ai_software_string)

replace author = author + " [" + ai_software_string + "]" if `software'

capture drop `var'_string

decode `var', gen(`var'_string)

replace `var'_string = "{bf:"+ `var'_string + "}"

if "`model'" == "" local model random

local lbe: variable label `var'

if "`title'" == "" local title = "{bf: `lbe'}, size(*0.8)"

sort `var'_string author

noisily list study_id `var' `var'_string tp fp fn tn

noisily metadta tp fp fn tn, studyid(author) sortby(`var') model(`model') dp(2) `download' sumtable(all) by(`var'_string) stratify ///

soptions(xtitle("False positive rate") bubbleid bubbles xlabel(0(0.2)1, format(%3.1f)) xscale(range(0 1)) ///

ytitle("Sensitivity") yscale(range(0 1)) ylabel(0(0.2)1,grid format(%3.1f)) ///

graphregion(color(white)) plotregion(margin(medium)) xsize(15) ysize(15)) ///

foptions(graphsave(metadta_`var') note(`note') title("") texts(`size') grid graphregion(color(white)) xlabel(0.5, 0.6, 0.7, 0.8, 0.9, 1) ///

diamopt(color(`color')) pointopt(msymbol(s)msize(1)) olineopt(color(`color') lpattern(dash)))

graph use metadta_`var'.gph

*** get rows of graph and resize

levelsof `var' , local(lev)

local lev_counter = 0

qui foreach l of local lev{

local ++lev_counter

su `var' if `var' == `l'

local numb = r(N)

if `numb'>1 local ++lev_counter

local lev_counter = `lev_counter' + `numb'

local ++lev_counter

}

local no_studies = `lev_counter'

local ysize = (3.865 + 7.52 / 17 * (`no_studies' +1)) /2.53

resize , ysize(`ysize') xsize(7.5)

local ysize_exp = round(`ysize'*500)

graph export `pdfname'.pdf, replace

rm metadta_`var'.gph

restore

}

end

*** PROG CREATES A DTA-FORESTPLOT BUT AGGREGATES THE STUDIES FIRST USING METADTA BY A SPECIFIED SUBGROUP AND SAVES IT TO PDF

capture prog drop by_aggr_group_forest

program by_aggr_group_forest

syntax, var(name) byvar(name) [model(string) pdfname(string) color(string) save_dta(integer 0) size(real 2) title(string) software(integer 0) note(string)]

version 18.0

qui{

preserve

if "`pdfname'" == "" local pdfname = `var'_forrest

keep if !missing(`var')

qui gen double dum_code = `var' * 100000 + `byvar'

qui levelsof dum_code, local(levels)

qui foreach l of local levels {

gen dummy = dum_code == `l'

su dummy

if r(sum) > 1 pooled_sens_spec, var(dummy) author(author) text("*")

drop dummy

}

drop dum_code

list `var' `byvar' tp fp fn tn

bygroup_forest, var(`byvar') pdfname(`pdfname') model(`model') color(`color') save_dta(`save_dta') size(`size') software(`software') note(`note') title(`title')

restore

}

end

*** PROG CREATES A DTA-FORESTPLOT BUT AGGREGATES THE STUDIES FIRST USING METADTA BY A SPECIFIED SUBGROUP AND COMPATES IT AND SAVES IT TO PDF

capture prog drop by_aggr_group_forest_comp

program by_aggr_group_forest_comp

syntax, var(name) byvar(name) [model(string) color(string) pdfname(string) save_dta(integer 0) size(real 2) title(string) software(integer 0) note(string) noteopt(string)]

version 18.0

qui{

preserve

if "`noteopt'" != "" local noteopt = ", " + `"`noteopt'"'

if "`pdfname'" == "" local pdfname = `var'_forrest

keep if !missing(`var')

qui gen dum_code = `var' * 100000 + `byvar'

qui levelsof dum_code, local(levels)

qui foreach l of local levels {

gen dummy = dum_code == `l'

su dummy

if r(sum) > 1 pooled_sens_spec, var(dummy) author(author) text("*")

drop dummy

}

drop dum_code

bysort study_id: drop if _N !=2

capture drop `byvar'string

decode `byvar', gen(`byvar'string)

gsort `byvar'string

local oppenent1 = `byvar'string[1]

gsort -`byvar'string

local oppenent2 = `byvar'string[1]

replace `byvar'string = "{bf:"+ `byvar'string + "}"

tab `byvar'string reader

noisily list `byvar'string reader tp fp fn tn

*** Get relative sens. + spez.

rename `byvar'string v1

metadta tp fp fn tn v1, studyid(author) comparative model(`model') sumtable(all) nofp nosroc

matrix rel = e(rrout)

local rel_sens_es: display %3.2f rel[2,1]

local rel_sens_lci: display %3.2f rel[2,5]

local rel_sens_uci: display %3.2f rel[2,6]

local rel_sens_p_val: display %4.3f rel[2,4]

if "`rel_sens_p_val'" == "0.000" local rel_sens_p_val = "<0.001"

else local rel_sens_p_val = "=`rel_sens_p_val'"

local rel_spec_es: display %3.2f rel[4,1]

local rel_spec_lci: display %3.2f rel[4,5]

local rel_spec_uci: display %3.2f rel[4,6]

local rel_spec_p_val: display %4.3f rel[4,4]

if "`rel_spec_p_val'" == "0.000" local rel_spec_p_val = "<0.001"

else local rel_spec_p_val = "=`rel_spec_p_val'"

local note_text = "{bf:`oppenent1' vs. `oppenent2':} Relative sensitivity: `rel_sens_es' (95% CI: `rel_sens_lci', `rel_sens_uci', p`rel_sens_p_val');"

local note_text2 = "Relative specificity: `rel_spec_es' (95% CI: `rel_spec_lci', `rel_spec_uci', p`rel_spec_p_val')"

sort author

noisily metadta tp fp fn tn v1, studyid(author) comparative model(`model') dp(2) `download' sumtable(all) ///

soptions(xtitle("False positive rate") bubbleid bubbles xlabel(0(0.2)1, format(%3.1f)) xscale(range(0 1)) ///

ytitle("Sensitivity") yscale(range(0 1)) ylabel(0(0.2)1,grid format(%3.1f)) ///

graphregion(color(white)) plotregion(margin(medium)) xsize(15) ysize(15)) ///

foptions(graphsave(metadta_comp_`byvar') note("`note_text'" "`note_text2'" "`note'" `noteopt') title("") texts(`size') grid graphregion(color(white)) xlabel(0.5, 0.6, 0.7, 0.8, 0.9, 1) diamopt(color(`color')) pointopt(msymbol(s)msize(1)) olineopt(color(`color') lpattern(dash)))

graph use metadta_comp_`byvar'.gph

levelsof `byvar' , local(lev)

local lev_counter = 0

qui foreach l of local lev{

local ++lev_counter

su `byvar' if `byvar' == `l'

local numb = r(N)

if `numb'>1 local ++lev_counter

local lev_counter = `lev_counter' + `numb'

local ++lev_counter

}

local no_studies = `lev_counter' + 2

local ysize = (3.865 + 7.52 / 17 * (`no_studies' +1)) /2.53

resize , ysize(`ysize') xsize(7.5)

local ysize_exp = round(`ysize'*500)

graph export `pdfname'.pdf, replace

rm metadta_comp_`byvar'.gph

restore

}

end

*** ESTIMATES THE POOLED TP; TN; TN; FN OUT OF THE POOLED SENS + SPEC OBTAINED BY METADTA

program pooled_sens_spec

version 18.0

syntax [if] [in], var(name) author(name) text(string)

qui {

capture metadta tp fp fn tn if `var' == 1, studyid(`author') nofp nosroc

if _rc == 0 {

matrix define A = e(absoutsp)

matrix define B = e(absoutse)

gen _orig = _n

bysort `var': drop if _n>1 & `var' == 1

replace author = author + "`text'" if `var' == 1

sort _orig

drop _orig

local cond = "if `var' == 1"

replace spec_es = A[1,1] `cond'

replace spec_lci = A[1,5] `cond'

replace spec_uci = A[1,6] `cond'

replace sens_es = B[1,1] `cond'

replace sens_lci = B[1,5] `cond'

replace sens_uci = B[1,6] `cond'

replace tp = round(0.5 * ((sens_es * (sens_es*(1-sens_es)) / ((sens_es-sens_lci)/1.96)^2)) + 0.5 * ((sens_es * (sens_es*(1-sens_es)) / ((sens_uci-sens_es)/1.96)^2))) `cond'

replace fn = round(0.5*(1-sens_es)*(sens_es*(1-sens_es)) / ((sens_es-sens_lci)/1.96)^2 + 0.5*(1-sens_es) * (sens_es*(1-sens_es)) / ((sens_uci-sens_es)/1.96)^2) `cond'

replace tn = round(0.5 * ((spec_es * (spec_es*(1-spec_es)) / ((spec_es-spec_lci)/1.96)^2) + (spec_es * (spec_es*(1-spec_es)) / ((spec_uci-spec_es)/1.96)^2))) `cond'

replace fp = round(0.5*(1-spec_es)*(spec_es*(1-spec_es)) / ((spec_es-spec_lci)/1.96)^2 + 0.5*(1-spec_es) * (spec_es*(1-spec_es)) / ((spec_uci-spec_es)/1.96)^2) `cond'

} // end if

} // end qui

end

NOTE: The raw dataset (filename: raw_dataset.dta) is available from the corresponding author by reasonable request
