# Supplementary material for: Artificial intelligence in commercial fracture detection products: a systematic review and meta-analysis of diagnostic test accuracy
Source: Sci Rep. 2024 Oct 4;14:23053. doi: 10.1038/s41598-024-73058-8 (PMC11452402; doi:10.1038/s41598-024-73058-8)
Supplement: Supplementary file 2 — Supplementary Material 2 [file 41598_2024_73058_MOESM2_ESM.docx]

# Supplement

## Supplement I. Stata code to calculate the diagnostic accuracy table

Estimation of true positives (tp) and false negatives (fn) out of sensitivity (sens_es) lower 95% CI (sens_lci) and upper 95% CI sens_uci).

replace tp = round(0.5 * ((sens_es * (sens_es*(1-sens_es)) / ((sens_es-sens_lci)/1.96)^2)) + 0.5 * ((sens_es * (sens_es*(1-sens_es)) / ((sens_uci-sens_es)/1.96)^2)))

replace fn = round(0.5*(1-sens_es)*(sens_es*(1-sens_es)) / ((sens_es-sens_lci)/1.96)^2 + 0.5*(1-sens_es) * (sens_es*(1-sens_es)) / ((sens_uci-sens_es)/1.96)^2)

Estimation of true negatives (tn) and false positives (fp) out of specificity (spec_es) lower 95% CI (spec_lci) and uppper 95% CI spec_uci).

replace tn = round(0.5 * ((spec_es * (spec_es*(1-spec_es)) / ((spec_es-spec_lci)/1.96)^2) + (spec_es * (spec_es*(1-spec_es)) / ((spec_uci-spec_es)/1.96)^2)))

replace fp = round(0.5*(1-spec_es)*(spec_es*(1-spec_es)) / ((spec_es-spec_lci)/1.96)^2 + 0.5*(1-spec_es) * (spec_es*(1-spec_es)) / ((spec_uci-spec_es)/1.96)^2)

## Supplement II. Additional forest plot analyses

#### Supplementary Figure 1. Diagnostic accuracy with 95% CI according to different body regions by human unaided and aided raters


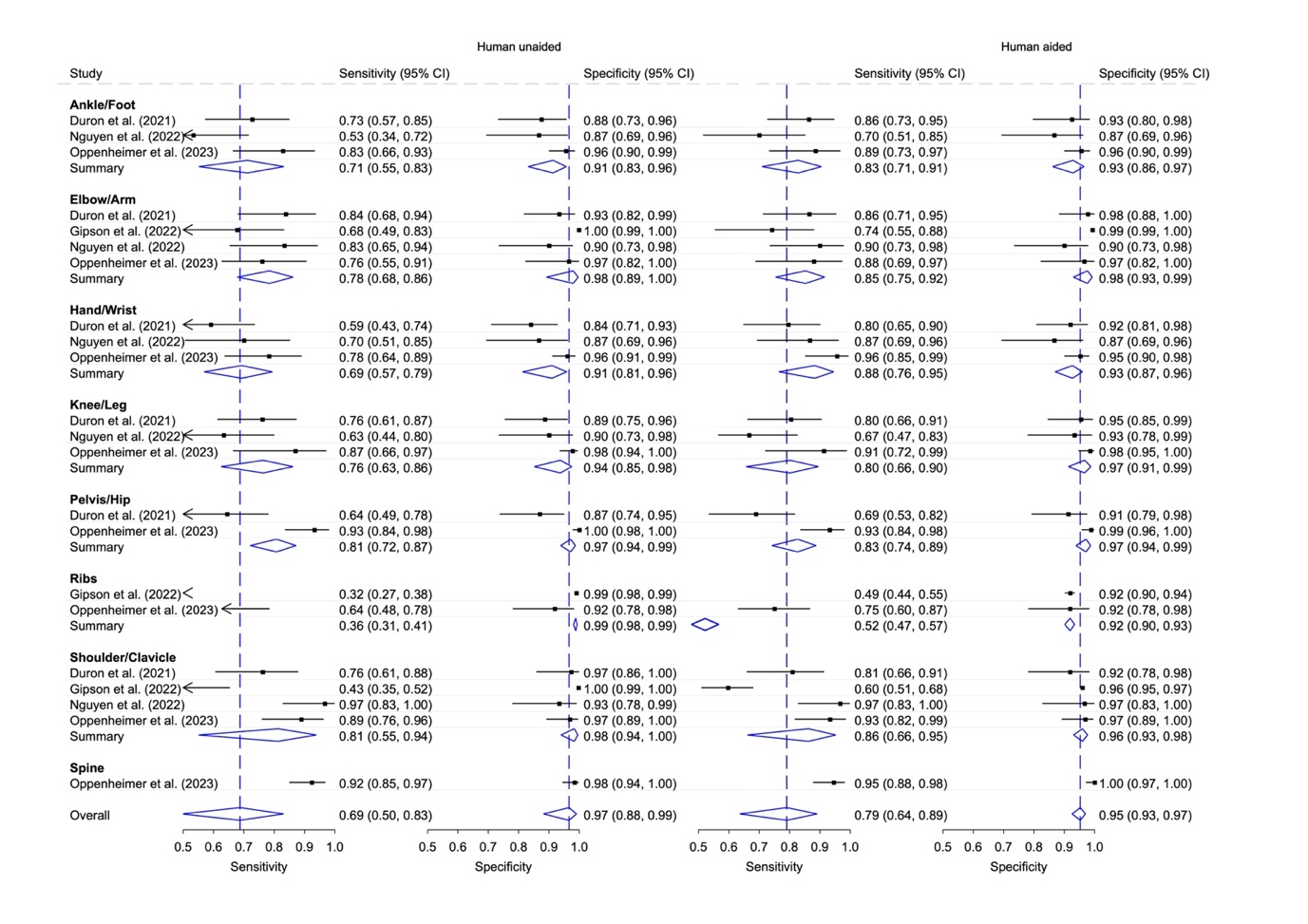


Generalized I^2^ *Unaided*: Ankle/Foot <0.01; Elbow/Arm <0.01; Hand/Wrist <0.01; Knee/Leg <0.01; Pelvis/Hip -; Ribs -; Shoulder/Clavicle <0.01; Spine -; Overall 0.24, respectively *Aided*: Ankle/Foot <0.01; Elbow/Arm <0.01; Hand/Wrist <0.01; Knee/Leg <0.01; Pelvis/Hip -; Ribs -; Shoulder/Clavicle <0.01; Spine -; Overall 0.17

#### Supplementary Figure 2. Diagnostic accuracy with 95% CI according to all type of raters (total)


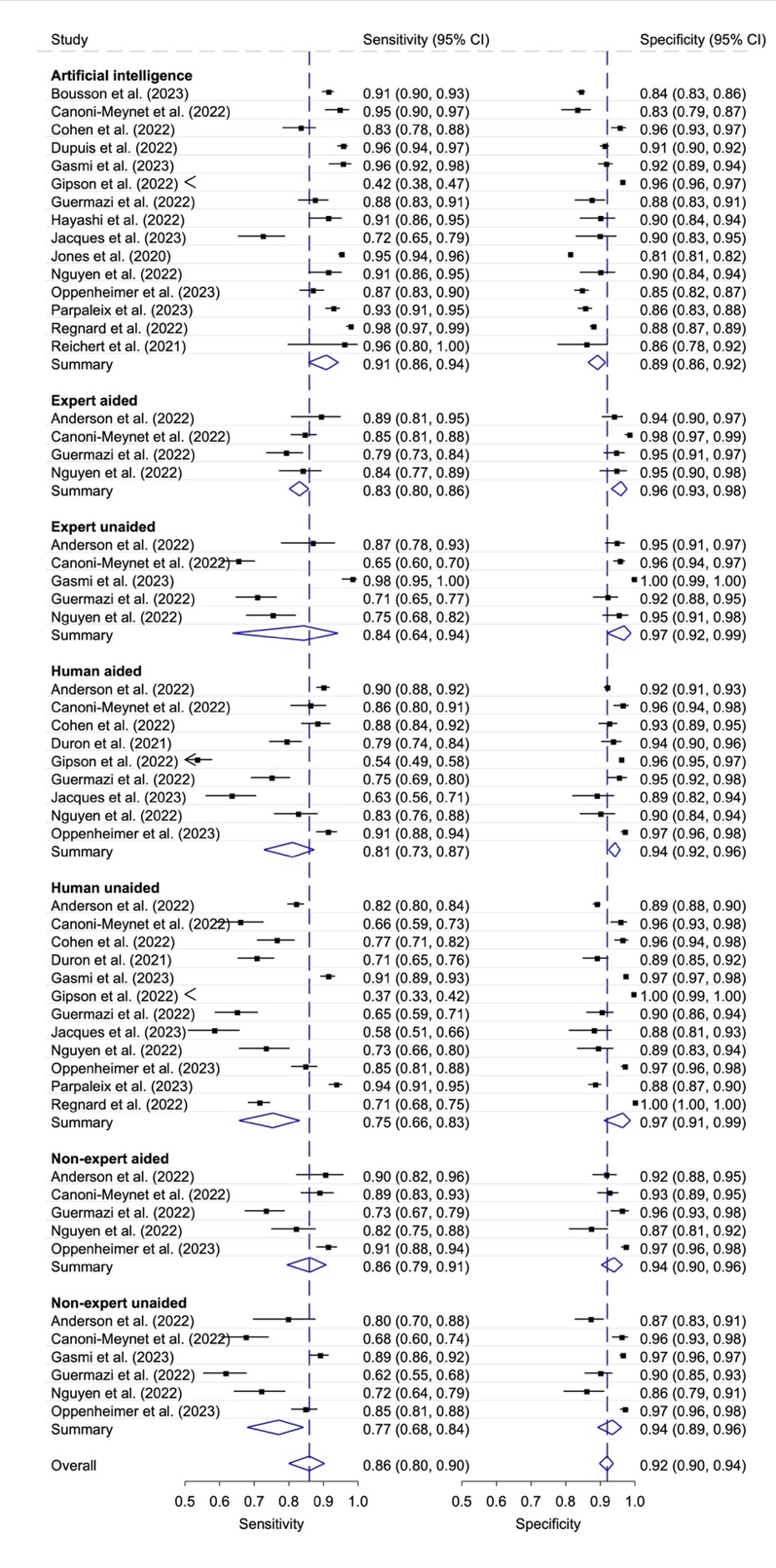


Generalized I^2^: Artificial intelligence 0.86; Expert aided <0.01; Expert unaided 0.77; Human aided 0.87; Human unaided 0.94; Non-expert aided 0.80; Non-expert unaided 0.86; Overall 0.70

#### Supplementary Figure 3. Diagnostic accuracy with 95% CI depending on the type of rater (stand-alone AI and human rater aided/unaided) excluding funded studies


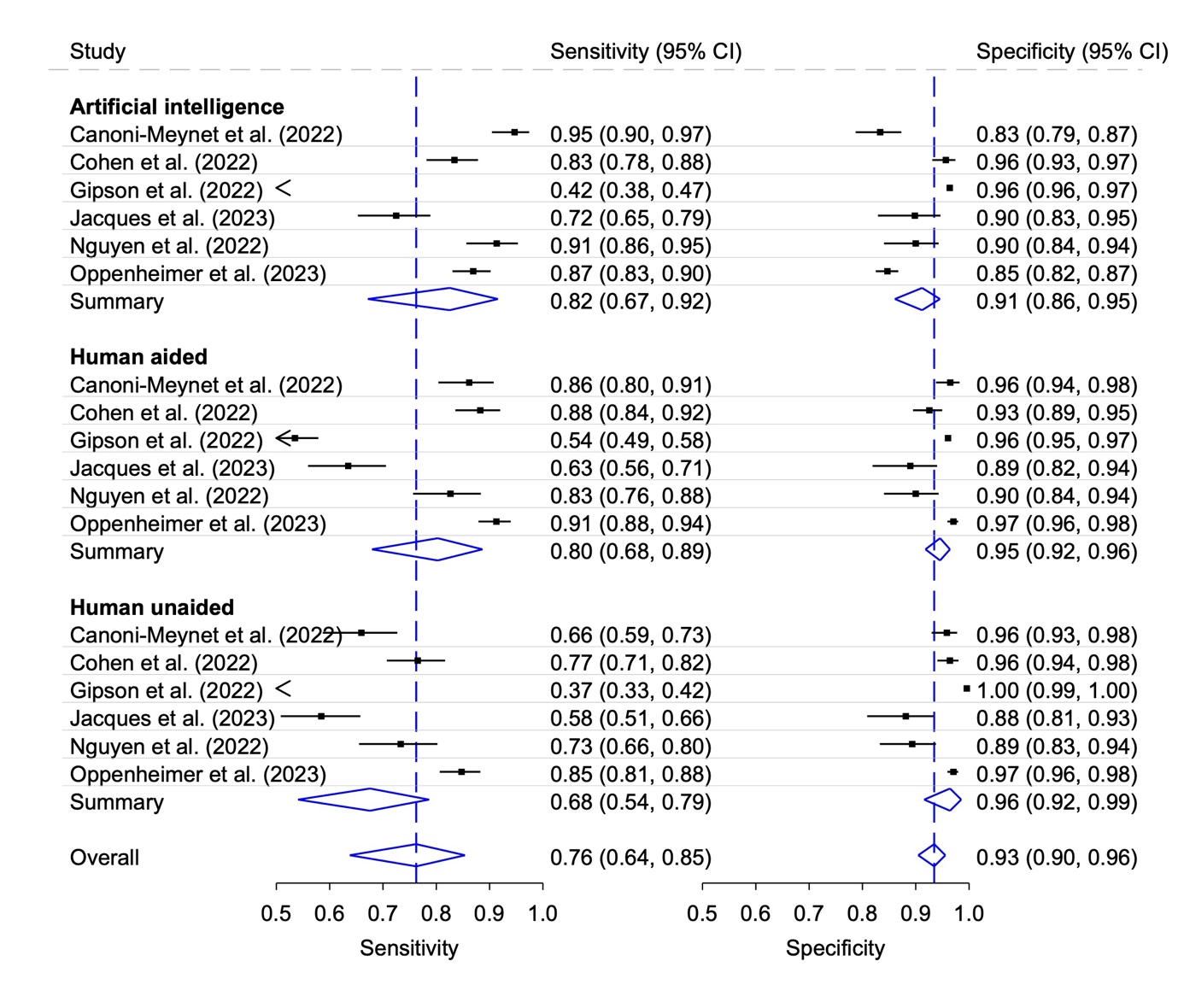


Generalized I^2^: Artificial intelligence 0.88; Human aided 0.88; Human unaided 0.91; Overall 0.69

## Supplement III. Search syntax for PubMed and Embase

("BoneView" OR "Gleamer") OR ("Rayvolve" OR "AZmed") OR ("SmartUrgences" OR "Milvue") OR ("Chest MSK" OR "Arterys") OR ("RBfracture" OR "Radiobotics") OR ("C-Spine (CSF)" OR "Rib fractures (RibFx)" OR "Aidoc") OR ("HealthOST" OR "Nanox") OR ("OsteoDetect" OR "FractureDetect" OR "Imagen Technologies") OR ("uAI Easytriage" OR "United Imaging") OR ("Enterprise CXR" OR "Annalise")

## Supplement IV. Signaling Questions Risk of Bias

Signalling questions for the concerns about Risk of Bias:

1. Patient selection

Was sufficient patient data provided (e.g. age, gender distribution, number of patient, and images)?

1. Index test

Were the index test results interpreted without knowledge of the results of the reference standard?

Since the AI does not know the reference standards, all included studies will receive a low Risk of Bias for this domain.

1. Reference standard

Was the reference standard mentioned and sufficiently described?

1. **REMOVED** Flow and timing

The original tool includes ‘Flow and Timing’ which describes the time interval and any interventions between index test(s) and reference standard. Patients in this review did not receive any interventions for which a time interval would be relevant, this domain was removed, and the funding status was included instead.

1. Funding

Was the study funded by the product vendor?

Signalling questions for the concerns about applicability:

1. Patient selection

Was the selection of patients applicable to the general research question of the review or was the patient selection not applicable as only children/adolescents or single body regions were assessed and thus considered to be of low applicability?

1. Index test

Are there any concerns that the index test, its administration or its interpretation deviates from the review question?

As the AI does not know the reference standard, all included studies are considered to have good applicability as they are commercially available software subject to certification.

1. Reference standard

Are there concerns that the target state defined by the reference standard does not appear to be sufficient (e.g. only one rater, assessment only by residents, exclusive use of reports)?

## Supplement V. Between-study heterogeneity statistics for all forest plots in the manuscript

| **Figure 2** |  |  |  |  |  |  |
| --- | --- | --- | --- | --- | --- | --- |
|  | **Sensitivity** | | **Specificity** | | **Generalized** | |
|  | **τ^2^** | **I^2^** | **τ^2^** | **I^2^** | **τ^2^** | **I^2^** |
| Artificial intelligence | 0.95 | 0.90 | 0.24 | 0.87 | 0.13 | 0.86 |
| Human aided | 0.49 | 0.94 | 0.17 | 0.72 | 0.08 | 0.87 |
| Human unaided | 0.69 | 0.97 | 3.06 | 0.90 | 1.95 | 0.94 |
|  |  |  |  |  |  |  |
| Overall | 0.73 | 0.87 | 0.28 | 0.75 | 0.14 | 0.79 |
|  |  |  |  |  |  |  |
|  |  |  |  |  |  |  |
| **Figure 3** |  |  |  |  |  |  |
|  | **Sensitivity** | | **Specificity** | | **Generalized** | |
|  | **τ^2^** | **I^2^** | **τ^2^** | **I^2^** | **τ^2^** | **I^2^** |
| BoneView | 0.54 | 0.90 | 0.12 | 0.75 | 0.06 | 0.82 |
| Enterprise CXR TT | - | - | - | - | - | - |
| FractureDetect | - | - | - | - | - | - |
| Rayvolve | 0.05 | 0.17 | 0.39 | 0.92 | <0.01 | <0.01 |
| SmartUrgence | <0.01 | <0.01 | <0.01 | <0.01 | - | - |
|  |  |  |  |  |  |  |
| Overall | 0.95 | 0.89 | 0.24 | 0.86 | 0.13 | 0.85 |
|  |  |  |  |  |  |  |
|  |  |  |  |  |  |  |
| **Figure 4** |  |  |  |  |  |  |
|  | **Sensitivity** | | **Specificity** | | **Generalized** | |
|  | **τ^2^** | **I^2^** | **τ^2^** | **I^2^** | **τ^2^** | **I^2^** |
| Ankle/Foot | 0.10 | 0.27 | 0.43 | 0.76 | <0.01 | <0.01 |
| Elbow/Arm | 1.91 | 0.75 | 1.14 | 0.72 | <0.01 | 0.01 |
| Hand/Wrist | 0.13 | 0.29 | 0.33 | 0.72 | <0.01 | <0.01 |
| Knee/Leg | 1.02 | 0.60 | 0.03 | 0.15 | <0.01 | 0.02 |
| Pelvis/Hip | 0.04 | 0.05 | 0.03 | 0.17 | <0.01 | <0.01 |
| Ribs | 0.58 | 0.85 | 0.60 | 0.77 | <0.01 | <0.01 |
| Shoulder/Clavicle | 1.33 | 0.69 | 0.32 | 0.57 | <0.01 | <0.01 |
| Spine | 0.65 | 0.68 | 0.09 | 0.59 | <0.01 | <0.01 |
|  |  |  |  |  |  |  |
| Overall | 1.00 | 0.28 | 0.29 | 0.22 | 0.13 | 0.18 |
|  |  |  |  |  |  |  |
|  |  |  |  |  |  |  |
| **Figure 5** |  |  |  |  |  |  |
|  | **Sensitivity** | | **Specificity** | | **Generalized** | |
|  | **τ^2^** | **I^2^** | **τ^2^** | **I^2^** | **τ^2^** | **I^2^** |
| Expert consensus | 0.17 | 0.74 | 0.21 | 0.86 | 0.02 | 0.73 |
| Others | 1.79 | 0.91 | 0.26 | 0.88 | 0.23 | 0.85 |
|  |  |  |  |  |  |  |
| Overall | 0.95 | 0.9 | 0.24 | 0.87 | 0.13 | 0.86 |
|  |  |  |  |  |  |  |
|  |  |  |  |  |  |  |
| **Figure 6** |  |  |  |  |  |  |
|  | **Sensitivity** | | **Specificity** | | **Generalized** | |
|  | **τ^2^** | **I^2^** | **τ^2^** | **I^2^** | **τ^2^** | **I^2^** |
| Industry funding | 0.44 | 0.86 | 0.07 | 0.73 | 0.03 | 0.8 |
| Other/no funding | 1.01 | 0.9 | 0.28 | 0.87 | 0.16 | 0.86 |
|  |  |  |  |  |  |  |
| Overall | 0.95 | 0.9 | 0.24 | 0.87 | 0.13 | 0.86 |
|  |  |  |  |  |  |  |
|  |  |  |  |  |  |  |
| **Figure 7** |  |  |  |  |  |  |
|  | **Sensitivity** | | **Specificity** | | **Generalized** | |
|  | **τ^2^** | **I^2^** | **τ^2^** | **I^2^** | **τ^2^** | **I^2^** |
| Low | 1.38 | 0.95 | 0.31 | 0.88 | 0.13 | 0.87 |
| Moderate | 0.25 | 0.86 | 0.21 | 0.90 | 0.04 | 0.87 |
| High | <0.01 | <0.01 | <0.01 | <0.01 |  |  |
|  |  |  |  |  |  |  |
| Overall | 0.95 | 0.90 | 0.24 | 0.87 | 0.13 | 0.86 |
|  |  |  |  |  |  |  |
|  |  |  |  |  |  |  |
| **Figure 8** |  |  |  |  |  |  |
|  | **Sensitivity** | | **Specificity** | | **Generalized** | |
|  | **τ^2^** | **I^2^** | **τ^2^** | **I^2^** | **τ^2^** | **I^2^** |
| Artificial intelligence | 0.91 | 0.95 | 0.33 | 0.87 | 0.09 | 0.86 |
| Human aided | 0.55 | 0.94 | 0.21 | 0.71 | 0.11 | 0.86 |
| Human unaided | 0.42 | 0.95 | 1.18 | 0.88 | 0.38 | 0.91 |
|  |  |  |  |  |  |  |
| Overall | 0.47 | 0.85 | 0.22 | 0.53 | 0.07 | 0.67 |
|  |  |  |  |  |  |  |
|  |  |  |  |  |  |  |
| **Supplementary Figure 1** | |  |  |  |  |  |
| **Unaided** |  |  |  |  |  |  |
|  | **Sensitivity** | | **Specificity** | | **Generalized** | |
|  | **τ^2^** | **I^2^** | **τ^2^** | **I^2^** | **τ^2^** | **I^2^** |
| Ankle/Foot | 0.24 | 0.61 | 0.20 | 0.40 | <0.01 | <0.01 |
| Elbow/Arm | 0.11 | 0.35 | 2.66 | 0.39 | <0.01 | <0.01 |
| Hand/Wrist | 0.10 | 0.45 | 0.34 | 0.54 | <0.01 | <0.01 |
| Knee/Leg | 0.15 | 0.44 | 0.42 | 0.49 | <0.01 | <0.01 |
| Pelvis/Hip | <0.01 | <0.01 | <0.01 | <0.01 |  |  |
| Ribs | <0.01 | <0.01 | <0.01 | <0.01 |  |  |
| Shoulder/Clavicle | 1.41 | 0.85 | 1.34 | 0.38 | <0.01 | <0.01 |
| Spine |  |  |  |  |  |  |
|  |  |  |  |  |  |  |
| Overall | 0.65 | 0.45 | 1.82 | 0.19 | 0.62 | 0.24 |
|  |  |  |  |  |  |  |
|  |  |  |  |  |  |  |
| **Aided** |  |  |  |  |  |  |
|  | **Sensitivity** | | **Specificity** | | **Generalized** | |
|  | **τ^2^** | **I^2^** | **τ^2^** | **I^2^** | **τ^2^** | **I^2^** |
| Ankle/Foot | 0.16 | 0.44 | 0.15 | 0.29 | <0.01 | <0.01 |
| Elbow/Arm | 0.15 | 0.34 | 0.81 | 0.36 | <0.01 | <0.01 |
| Hand/Wrist | 0.27 | 0.49 | 0.07 | 0.19 | <0.01 | <0.01 |
| Knee/Leg | 0.21 | 0.49 | 0.27 | 0.26 | <0.01 | <0.01 |
| Pelvis/Hip | <0.01 | <0.01 | <0.01 | <0.01 |  |  |
| Ribs | <0.01 | <0.01 | <0.01 | <0.01 |  |  |
| Shoulder/Clavicle | 1.09 | 0.79 | <0.01 | <0.01 | <0.01 | <0.01 |
| Spine |  |  |  |  |  |  |
|  |  |  |  |  |  |  |
| Overall | 0.59 | 0.36 | 0.18 | 0.07 | 0.10 | 0.17 |
|  |  |  |  |  |  |  |
|  |  |  |  |  |  |  |
| **Supplementary Figure 2** | |  |  |  |  |  |
|  | **Sensitivity** | | **Specificity** | | **Generalized** | |
|  | **τ^2^** | **I^2^** | **τ^2^** | **I^2^** | **τ^2^** | **I^2^** |
| Artificial intelligence | 0.95 | 0.90 | 0.24 | 0.87 | 0.13 | 0.86 |
| Expert aided | <0.01 | 0.09 | 0.23 | 0.66 | <0.01 | <0.01 |
| Expert unaided | 1.52 | 0.95 | 1.15 | 0.85 | 0.20 | 0.77 |
| Human aided | 0.49 | 0.94 | 0.17 | 0.72 | 0.08 | 0.87 |
| Human unaided | 0.69 | 0.97 | 3.06 | 0.90 | 1.95 | 0.94 |
| Non-expert aided | 0.23 | 0.81 | 0.31 | 0.80 | 0.07 | 0.80 |
| Non-expert unaided | 0.30 | 0.90 | 0.43 | 0.87 | 0.09 | 0.86 |
|  |  |  |  |  |  |  |
| Overall | 0.76 | 0.80 | 0.31 | 0.65 | 0.17 | 0.70 |
|  |  |  |  |  |  |  |
|  |  |  |  |  |  |  |
| **Supplementary Figure 3** | |  |  |  |  |  |
|  | **Sensitivity** | | **Specificity** | | **Generalized** | |
|  | **τ^2^** | **I^2^** | **τ^2^** | **I^2^** | **τ^2^** | **I^2^** |
| Artificial intelligence | 1.04 | 0.96 | 0.38 | 0.88 | 0.13 | 0.88 |
| Human aided | 0.63 | 0.95 | 0.24 | 0.75 | 0.15 | 0.88 |
| Human unaided | 0.49 | 0.95 | 1.22 | 0.87 | 0.44 | 0.91 |
|  |  |  |  |  |  |  |
| Overall | 0.55 | 0.86 | 0.24 | 0.55 | 0.09 | 0.69 |
